# Supplementary material for: Joint genetic analysis using variant sets reveals polygenic gene-context interactions
Source: PLoS Genet. 2017 Apr 20;13(4):e1006693. doi: 10.1371/journal.pgen.1006693 (PMC5398484; doi:10.1371/journal.pgen.1006693)
Supplement: S1 Text — Derivation and implementation details of the gene-context interaction set tests. (PDF) [file pgen.1006693.s001.pdf]

Supplementary Methods: Joint genetic analysis using variant sets reveals polygenic gene-context interactions

Francesco Paolo Casale, Danilo Horta, Barbara Rakitsch, Oliver Stegle

In this supplementary document we provide a complete derivation and implementation details of the set tests for gene-context (GxC) interactions (iSet). In Section 1, we describe the model for the case of fully observed designs, when phenotype data are available in all contexts for every individual, and we contrast iSet to related tests for interactions with context. In Section 2 we discuss extensions of iSet to enable interaction analyses of stratified population using a context variable, where phenotype data for each individual are observed in only one of the contexts. Additionally, we provide implementation details of an efficient inference scheme for this setting. Finally, in Section 3 we provide additional details on the simulation framework.

1. Interaction set test

In this section we provide full details of iSet and compare the model to alternative interaction tests. In Section 1.1, we derive alternative covariance models from a generative linear model perspective considering complete data designs; in Sections 1.2 and 1.3 we discuss the statistical tests considered in iSet and the approach used for variance decomposition. In Section 1.4 we discuss the scalability of iSet for different designs and sample structures. Finally, in Section 1.5 we compare iSet to related interaction tests.

1.1. The model

We here derive the iSet model from a generative linear model perspective, initially considering a random effect to correct for relatedness and/or population structure. As discussed in Section 1.1.1, population structure can alternatively be modeled using fixed effect covariates.

The $N \times C$ phenotype matrix $\boldsymbol{Y}$ for N individuals and two or more contexts C is modelled as the sum of the contribution from K fixed effect covariates, the contribution from R variants in the region of interest (set component), a term accounting for population structure or relatedness (relatedness component) and residual noise

$$\boldsymbol{Y} = \underbrace{\boldsymbol{F}\boldsymbol{B}}_{\text{fixed effects}} + \underbrace{\boldsymbol{G}\boldsymbol{W}}_{\text{set component}} + \underbrace{\boldsymbol{U}_g}_{\text{relatedness component}} + \underbrace{\boldsymbol{\Psi}}_{\text{noise}}. \quad (1)$$

Here, $\boldsymbol{G} \in \mathbb{R}^{N \times R}$ denotes the standardised genotype matrix of the R genetic variants in the set of interest, $\boldsymbol{W} \in \mathbb{R}^{R \times C}$ the matrix of their effect sizes across the C contexts, $\boldsymbol{F} \in \mathbb{R}^{N \times K}$ the design matrix of K covariates, $\boldsymbol{B} \in \mathbb{R}^{K \times C}$ the matrix of their effect sizes, and $\boldsymbol{U}_g$ and $\boldsymbol{\Psi}$ are random effects that account for relatedness and residual noise, and follow matrix-variate normal distributions:

$$\boldsymbol{U}_g \sim \mathrm{MVN}\left(\boldsymbol{0}, \boldsymbol{C}_g, \boldsymbol{R}\right) \quad \text{and} \quad \boldsymbol{\Psi} \sim \mathrm{MVN}\left(\boldsymbol{0}, \boldsymbol{C}_n, \boldsymbol{I}_N\right), \quad (2)$$

where $\boldsymbol{R} \in \mathbb{R}^{N \times N}$ denotes the realized relatedness matrix (RRM) [1] and $\boldsymbol{C}_g$ and $\boldsymbol{C}_n$ are $C \times C$ covariance matrices accounting for co-variation of trait measurements across contexts due to the contributions from relatedness and noise, respectively.

Considering a normal prior on the effect sizes of each variant in the region across the analyzed contexts contexts

$$\boldsymbol{W}_{r,:} \sim \mathcal{N}\left(\boldsymbol{0}, \frac{1}{R}\boldsymbol{C}_s\right), \quad \forall r \in \{1, \ldots, R\} \tag{3}$$

where $\boldsymbol{C}_s$ is a $C \times C$ covariance matrix, and marginalizing out $\boldsymbol{W}$, we obtain the following marginal likelihood:

$$\text{vec}(\boldsymbol{Y}) \sim \mathcal{N}\left(\underbrace{\text{vec}(\boldsymbol{F}\boldsymbol{B})}_{\text{fixed effect covariates}}, \underbrace{\boldsymbol{C}_s \otimes \boldsymbol{R}_s}_{\text{set component}} + \underbrace{\boldsymbol{C}_g \otimes \boldsymbol{R}_g}_{\text{relatedness}} + \underbrace{\boldsymbol{C}_n \otimes \boldsymbol{I}_N}_{\text{noise}}\right) \tag{4}$$

where $\boldsymbol{R}_s = \frac{1}{R}\boldsymbol{G}\boldsymbol{G}^T \in \mathbb{R}^{N \times N}$ is the set (local) realized relatedness matrix (accounting for local genetic similarity) and $\boldsymbol{C}_s$ accounts for the co-variation of trait measurements across contexts due to the variants in the set. This model has previously been considered in [2] to test for associations between sets of variants and multiple quantitative traits and is amenable to fast inference (**Supplementary Figure 1**, **Supplementary Table 7**).

A key insight derived here is that different local architectures correspond to alternative assumptions on the structure of the trait-context covariance $\boldsymbol{C}_s$ (Figure 1). In the following, we show how alternative covariance structures for $\boldsymbol{C}_s$ correspond to different assumptions on the local genetic architecture. To simplify the notation, we consider the case of two contexts ($C = 2$). However, the derivation holds when more than two contexts are considered.

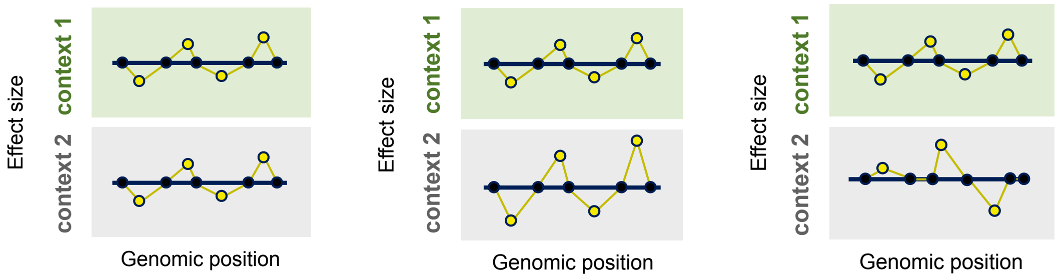

(a) Persistent effects **(b)** Rescaling-GxC effects **(c)** Heterogeneity-GxC effects

Figure 1: Schematic representation of alternative GxC architectures (x-axis: genomic position, y-axis: effect size). Yellow (dark) beads correspond to causal (non-causal) variants.

Persistent effect model. For local persistent effects, the matrix of the variant effect sizes can be written as

$$\boldsymbol{W} = [a\boldsymbol{\gamma}, a\boldsymbol{\gamma}] = \boldsymbol{\gamma} a \boldsymbol{1}_2^\top, \tag{5}$$

where the effect size profile $\boldsymbol{\gamma} \in \mathbb{R}^R$ of the genetic variants has same scale a in both contexts (Figure 1a). Considering the prior $\boldsymbol{\gamma} \sim \mathcal{N}(0, \frac{1}{R}\boldsymbol{I}_R)$ and marginalising out $\boldsymbol{\gamma}$, we obtain the model in Eq. (10) with $\boldsymbol{C}_s = a^2 \boldsymbol{1}_{2\times 2}$ (block covariance). One way to derive this result is to write $\boldsymbol{r} = \text{vec}(\boldsymbol{G}\boldsymbol{W}) =$

$\text{vec}\left(\boldsymbol{G}\boldsymbol{\gamma}a\mathbf{1}_2^\top\right) = a\left(\mathbf{1}_2 \otimes \boldsymbol{G}\right)\boldsymbol{\gamma}$; then from $\boldsymbol{\gamma} \sim \mathcal{N}(0, \frac{1}{R}\boldsymbol{I}_R)$, it follows

$$\boldsymbol{r} \sim \mathcal{N}\left(\mathbf{0}, \underbrace{a^2\mathbf{1}_2\mathbf{1}_2^\top}_{\boldsymbol{C}_s} \otimes \underbrace{\frac{1}{R}\boldsymbol{G}\boldsymbol{G}^\top}_{\boldsymbol{R}_s}\right). \tag{6}$$

Rescaling-GxC model. In the scenario of rescaling-GxC effects (Figure 1b), we consider

$$\boldsymbol{W} = [a_1\boldsymbol{\gamma}, a_2\boldsymbol{\gamma}] = \boldsymbol{\gamma}\boldsymbol{a}^\top, \tag{7}$$

where the effect size profile $\boldsymbol{\gamma} \in \mathbb{R}^R$ of the genetic variants has context-specific scales $\boldsymbol{a} = [a_1, a_2]$. Considering the prior $\boldsymbol{\gamma} \sim \mathcal{N}(\mathbf{0}, \frac{1}{R}\boldsymbol{I}_R)$ and marginalising $\boldsymbol{\gamma}$ out results in $\boldsymbol{C}_s = \boldsymbol{a}\boldsymbol{a}^\top$ (rank-one covariance).

This model can capture three distinct settings:

- $a_1a_2 > 0$, the genetic signal from the set has same direction;
- $a_1a_2 < 0$, the genetic signal from the set has opposite direction;
- $a_1 \approx 0$ and $a_2 \neq 0$ (or vice versa), the genetic signal from the set is specific to one of the two contexts.

Moreover, note that for $a_1 = a_2$ the model reduces to the persistent effect model.

General-GxC model. For the most general case in Figure 1c, we introduce effect size profiles $\boldsymbol{\gamma}^{(1)} \in \mathbb{R}^R$ and $\boldsymbol{\gamma}^{(2)} \in \mathbb{R}^R$ and set

$$\boldsymbol{W} = \left[a_{11}\boldsymbol{\gamma}^{(1)} + a_{12}\boldsymbol{\gamma}^{(2)},\ a_{21}\boldsymbol{\gamma}^{(1)} + a_{22}\boldsymbol{\gamma}^{(2)}\right] = \left[\boldsymbol{\gamma}^{(1)}, \boldsymbol{\gamma}^{(2)}\right]\boldsymbol{A}^\top, \tag{8}$$

where $\boldsymbol{A} = \begin{bmatrix} a_{11} & a_{12} \\ a_{21} & a_{22} \end{bmatrix}$ is a rescaling matrix (a_{ij} is the scale of variant effect profile $\boldsymbol{\gamma}_j$ in context i). Introducing the prior $\boldsymbol{\gamma}^{(1)}, \boldsymbol{\gamma}^{(2)} \sim \mathcal{N}(0, \frac{1}{R}\boldsymbol{I}_R)$ and marginalising $\boldsymbol{\gamma}^{(1)}$ and $\boldsymbol{\gamma}^{(2)}$ out, we obtain $\boldsymbol{C}_s = \boldsymbol{A}\boldsymbol{A}^\top$ (full-rank covariance). Note that the model includes both rescaling-GxC and persistent effects as special cases.

Analyses across more than two contexts. For analyses of $C > 2$ contexts we can consider a model with $L \leq C$ distinct genetic signals $\left\{\boldsymbol{\gamma}^{(1)}, \ldots, \boldsymbol{\gamma}^{(\mathrm{L})}\right\}$. Introducing $\boldsymbol{\Gamma}_L = \left[\boldsymbol{\gamma}^{(1)}, \ldots, \boldsymbol{\gamma}^{(\mathrm{L})}\right] \in \mathbb{R}^{R \times L}$ and the rescaling matrix $\boldsymbol{A}_L \in \mathbb{R}^{L \times C}$, we can write

$$\boldsymbol{W} = \boldsymbol{\Gamma}_L\boldsymbol{A}_L. \tag{9}$$

Setting $\boldsymbol{\Gamma}_L \overset{\text{iid}}{\sim} \prod \mathcal{N}(0, 1)$ we obtain $\boldsymbol{C}_s = \boldsymbol{A}_L\boldsymbol{A}_L^\top$, which has rank L. The rank of $\boldsymbol{C}_s$ is the number of distinct local genetic signals across the different contexts. Note that each signal may be polygenic, resulting from the joint effect of multiple variants in the set.

1.1.1. Using principal components to account for population structure

As discussed in [2], for the analysis of cohorts with unrelated individuals, population structure can be accounted for by including the leading principal components of the realized relatedness matrix as fixed effect covariates into the model. The model can be cast as

$$\text{vec}(\boldsymbol{Y}) \sim \mathcal{N}\left(\underbrace{\text{vec}(\boldsymbol{F}\boldsymbol{B})}_{\text{fixed effect covariates}}, \underbrace{\boldsymbol{C}_s \otimes \boldsymbol{G}\boldsymbol{G}^T}_{\text{set component}} + \underbrace{\boldsymbol{C}_n \otimes \boldsymbol{I}_N}_{\text{noise}}\right), \tag{10}$$

and it is amenable to even faster computations that scale linearly in the number of individuals (**Supplementary Figure 1**, **Supplementary Table 7**, see also [2]). We used this model for the analyses presented in the manuscript.

1.2. Statistical testing

Model comparisons of the LMM in Eq. (10) considering alternative covariance structures for the set trait covariance allows testing for different hypothesis on the local genetic architecture. Specifically, we consider the following tests:

- **Association test (mtSet).** The full-rank covariance model is tested against a null covariance model (no association):
$$\mathcal{H}_1 : \boldsymbol{C}_s = \boldsymbol{A}\boldsymbol{A}^\top \quad \text{vs} \quad \mathcal{H}_0 : \boldsymbol{C}_s = \boldsymbol{0} \tag{11}$$
- **Interaction test (iSet).** The full-rank covariance model is tested against a block covariance model (which only captures persistent effects):
$$\mathcal{H}_1 : \boldsymbol{C}_s = \boldsymbol{A}\boldsymbol{A}^\top \quad \text{vs} \quad \mathcal{H}_0 : \boldsymbol{C}_s = a^2\mathbf{1}_{2\times 2} \tag{12}$$
- **Heterogeneity-GxC test (iSet-het).** The full-rank covariance model is tested against a rank-one covariance model (which captures both persistent and rescaling-GxC effects):
$$\mathcal{H}_1 : \boldsymbol{C}_s = \boldsymbol{A}\boldsymbol{A}^\top \quad \text{vs} \quad \mathcal{H}_0 : \boldsymbol{C}_s = \boldsymbol{a}\boldsymbol{a}^\top \tag{13}$$

Note that the covariance models introduced in the previous section are nested and thus statistical tests are well defined. A summary of the different covariance structures considered by the model and their use to derive specific tests is shown in main paper Figure 1.

P values. For the proposed tests, we consider the log-likelihood ratio (LLR) test statistics. While for the standard association test (mtSet) P values can be obtained using a permutation approach [2], permutation schemes are not defined for interaction tests [3]. Following [3], we instead consider a parametric bootstrap procedure to estimate P values for the iSet and the iSet-het tests. This procedure is based on generating test statistics from an empirical null by drawing phenotypes from the null model with parameter values that maximize the likelihood on real data. Similar to the strategy employed for mtSet, we consider a small number of parametric bootstraps for each region (typically 30 bootstraps) and pool the obtained null LLRs across all tested regions. We then use the estimated distribution of null LLRs to obtain empirical P values.

In an analysis of T genomic regions, the procedure to obtain P values for the the three tests can be summarised as follows:

- For each of the T sets
  - fit the no-association model ($\mathcal{H}_{\text{na}}$), the block covariance model ($\mathcal{H}_{\text{block}}$), the rank-one covariance model ($\mathcal{H}_{\text{rank1}}$) and the full-rank covariance model ($\mathcal{H}_{\text{full}}$) and estimate LLRs for mtSet ($\mathcal{H}_{\text{full}}$ vs $\mathcal{H}_{\text{na}}$), iSet ($\mathcal{H}_{\text{full}}$ vs $\mathcal{H}_{\text{block}}$) and iSet-het ($\mathcal{H}_{\text{full}}$ vs $\mathcal{H}_{\text{rank1}}$);
  - sample J LLRs from the null hypothesis for each of the three tests
    - for mtSet, null LLRs are sampled as the LLRs from the mtSet test considering J permutations the individuals in the set component;
    - for iSet, null LLRs are sampled as the LLRs from the iSet test considering J parametric bootstraps from $\mathcal{H}_{\text{block}}$;

* for iSet-het, null LLRs are sampled as the LLRs from the iSet-het test considering J parametric bootstraps from $\mathcal{H}_{\mathrm{rank1}}$;

- for each of the three tests, pool the JT null LLRs across regions to obtain an empirical null. Empirical P values are obtained as the fraction of null LLRs that are at least as extreme as the observed one.

1.3. Interpretation of the variance parameters

The expected sample variance of a random vector $\boldsymbol{u}$ that follows a multivariate normal distribution with mean $\mathbf{0}$ and covariance $\boldsymbol{K}$, $\boldsymbol{u} \sim \mathcal{N}(\mathbf{0}, \mathbf{K})$, is (see [4], p 67)

$$\mathbb{E}\left[\operatorname{var}(\boldsymbol{u})\right] = \frac{\operatorname{tr}(\boldsymbol{P}_n \boldsymbol{K})}{n-1}, \tag{14}$$

where n is the total number of samples and $\boldsymbol{P}_n = \boldsymbol{I}_n - \frac{1}{n}\mathbf{1}_{n\times n}$ is the centring matrix. Using Eq. (14), iSet allows for estimating (i) the fraction of variance explained by the genetic region [5] and (ii) the relative proportions of local variance that is explainable by persistent, rescaling-GxC and heterogeneity-GxC effects (Figure 1d, main).

Fraction of variance explained by the region. Considering the model in Eq. (10), the variance explained by the set component (v_{set}), the relatedness (v_{rel}) and the noise component (v_{noise}) across all observations are

$$\begin{aligned} v_{\mathrm{set}} &= \frac{1}{NC-1}\operatorname{tr}\left(\boldsymbol{P}_n\left(\hat{\boldsymbol{C}}_s \otimes \boldsymbol{R}_s\right)\right) && (15)\\ v_{\mathrm{rel}} &= \frac{1}{NC-1}\operatorname{tr}\left(\boldsymbol{P}_n\left(\hat{\boldsymbol{C}}_g \otimes \boldsymbol{R}_g\right)\right) && (16)\\ v_{\mathrm{noise}} &= \frac{1}{NC-1}\operatorname{tr}\left(\boldsymbol{P}_n\left(\hat{\boldsymbol{C}}_n \otimes \boldsymbol{I}_N\right)\right). && (17)\end{aligned}$$

Here $\hat{\boldsymbol{C}}_s$, $\hat{\boldsymbol{C}}_g$ and $\hat{\boldsymbol{C}}_n$ denote the maximum-likelihood estimator (MLE) of $\boldsymbol{C}_s$, $\boldsymbol{C}_g$ and $\boldsymbol{C}_n$ considering a full-rank covariance model for $\boldsymbol{C}_s$. The expected proportion of variance explained by the genetic region can be estimated as

$$h^2_{\mathrm{set}} = \frac{v_{\mathrm{set}}}{\operatorname{var}(\operatorname{vec}(\boldsymbol{FB})) + v_{\mathrm{set}} + v_{\mathrm{rel}} + v_{\mathrm{noise}}}. \tag{18}$$

When population structure is modelled using fixed effects, we have $v_{\mathrm{rel}} = 0$ and the contribution to variance of population structure is in $\operatorname{var}(\operatorname{vec}(\boldsymbol{FB}))$. Note that v_{set}, v_{rel} and v_{noise} can be computed efficiently by exploiting that $\boldsymbol{P}_{NC}$ can be written as sum of Kronecker products:

$$\boldsymbol{P}_{NC} = \boldsymbol{I}_{NC} - \frac{1}{NC}\mathbf{1}_{NC\times NC} = \boldsymbol{I}_C \otimes \boldsymbol{I}_N - \frac{1}{NC}\left(\mathbf{1}_{C\times C} \otimes \mathbf{1}_{N\times N}\right). \tag{19}$$

Decomposing the local variance. To estimate the relative proportions of local variance that is explainable by persistent, rescaling-GxC and heterogeneity-GxC effects we use the following strategy:

- consider the block and the low-rank approximation of $\hat{\boldsymbol{C}}_s$

$$\begin{aligned} \hat{\boldsymbol{C}}_s^{(\mathrm{block})} &= \operatorname{mean}(\hat{\boldsymbol{C}}_s)\mathbf{1}_{C\times C} && (20)\\ \hat{\boldsymbol{C}}_s^{(\mathrm{lr})} &= \lambda \boldsymbol{v}\boldsymbol{v}^{\top}, && (21)\end{aligned}$$

  where λ is the largest eigenvalue of $\hat{\boldsymbol{C}}_s$ and $\boldsymbol{v}$ the corresponding eigenvector;

- use Eq. (14) to estimate the expected sample variance of the random vectors $\boldsymbol{u}^{(\text{block})} \sim \mathcal{N}\left(\mathbf{0}, \hat{\boldsymbol{C}}_s^{(\text{block})} \otimes \boldsymbol{R}_s\right)$ and $\boldsymbol{u}^{(\text{lr})} \sim \mathcal{N}\left(\mathbf{0}, \hat{\boldsymbol{C}}_s^{(\text{lr})} \otimes \boldsymbol{R}_s\right)$, which we denote with v_{pers} and v_{lr} respectively;
- define the variance explained by persistent, rescaling-GxC and heterogeneity-GxC as v_{pers}, $v_{\text{lr}} - v_{\text{pers}}$, $v_{\text{set}} - v_{\text{lr}}$ [1] .

1.4. Data design, sample relatedness and scalability

A naive implementation of the set test in Eq. (1) would scale cubically with the number of individuals and contexts. First, in case of fully observed phenotype data we re-use the efficient inference scheme proposed in [2], reducing the computational complexity from $O(tN^3)$ to $O(N^3 + N^2R + tNR^2)$, where N denotes the number of samples, R the number of variants in the region and t the number of likelihood and gradient evaluations which is necessary for optimization. This speed-up is effective if $R < N$. As discussed in Section 1.1.1, in analyses of unrelated samples, population structure can be modeled by introducing the top principal components of the realized relationship matrix as fixed effect covariates rather than considering a random effect. This further reduces computational complexity to $O(NR^2 + tNN_{\text{pc}}^2)$ where N_{pc} denotes the number of principal components. In the next section, we discuss the extension of the efficient algorithm for the analysis of unrelated individuals to handle incomplete data designs, in which phenotype data for any sample are observed only in one of the analyzed contexts, which reduces computational complexity to $O(t(NR^2 + NN_{\text{pc}}^2))$. This extension enables applications of iSet to analyses in stratified samples from large GWAS cohorts. A tabular summary of the complexities of iSet in the different analysis scenarios is given in **Supplementary Table 7**. In **Supplementary Fig. 1** we show the empirical CPU time considering increasingly sized simulated cohorts.

1.5. Comparison with related interaction tests

iSet extends existing multivariate LMMs [6, 7] and set-based interaction tests [8, 9, 10, 11, 12] (see Figure 2 for a tabular comparison). While multivariate LMMs have been limited to analyses of single variants, existing interaction set tests build on a univariate LMM and cannot be applied to analyze datasets with complete designs. In the following, we provide a brief overview of these methods.

Method	Paper	LMM type	Multiple variants	Fully observed designs	Stratified samples	Signal heterogeneity across contexts	Variance decomposition	Variant type
**iSet**	-	multivariate	✓	✓	✓	✓	✓	common
**MTMM**	*Korte et al (2012)*	multivariate	✗	✓	✗	✓	✗	common
**GESAT / iSKAT**	*Lin et al (2013)*<br>*Lin et al (2016)*	univariate	✓	✗	✓	✗	✗	common / rare
**SimReg**	*Tzeng et al (2011)*<br>*Zhao et al (2015)*	univariate	✓	✗	✓	✗	✗	common / rare
**Turkey's 1dof test**	*Chatterjee et al (2006)*	univariate	✓	✗	✓	✗	✗	common

Figure 2: **Comparison table of iSet and related models for interaction testing.**

Multi-trait LMMs for interaction test. We here describe the multi-trait linear mixed model (mtLMM) proposed in [6], which allows testing for association and interaction across multiple contexts in analyses

[1] Note that the defined variances are well defined (i.e., non-negative) as $v_{\text{pers}} \leq v_{\text{lr}} \leq v_{\text{set}}$.

of datasets with complete designs. Using the notation in Section 1.1 and indicating with $\boldsymbol{g} \in \mathbb{R}^N$ and $\boldsymbol{w} \in \mathbb{R}^C$ the genotype of the variant being tested and its effect sizes in the different contexts, the mtLMM model can be written as

$$\boldsymbol{Y} = \underbrace{\boldsymbol{F}\boldsymbol{B}}_{\text{fixed effect covariates}} + \underbrace{\boldsymbol{g}\boldsymbol{w}^T}_{\text{genetic variant effect}} + \underbrace{\boldsymbol{U}_g}_{\text{relatedness effect}} + \underbrace{\boldsymbol{\Psi}}_{\text{noise}}, \tag{22}$$

Association and GxC interaction tests can be performed respectively by testing $\boldsymbol{w} \neq \boldsymbol{0}$ and $\boldsymbol{w} \neq w_0\boldsymbol{1}$. In our implementation of the model, we use the log likelihood ratio (LLR) test statistics for both tests. Following [6], we estimate variance components on the no-association model ($\boldsymbol{w} = \boldsymbol{0}$) and update only the total variance in single variant testing. Zhou et al. [13] proposed an efficient inference scheme to re-fit the variance components for each tested variant when testing for association, however do not consider interaction tests. A more detailed description of the model and its implementation can be found in [6, 14]. iSet generalizes mtLMM by modeling effects from multiple genetic variants. In Section 2.4, we describe the extension of mtLMM for analysis of stratified samples, which we considered as comparison partner in both simulated and real data analyses of stratified samples.

Set-based interaction tests. One of the first set tests is the Turkey's one degree-of-freedom (dof) test [8]. Using the notation introduced above and denoting with $\{\boldsymbol{g}_1, \dots \boldsymbol{g}_R\}$ the genotype vectors for the R variants in the analysed region, [8] considered the model

$$\boldsymbol{y} = \underbrace{\boldsymbol{F}\boldsymbol{b}}_{\text{covariates}} + \underbrace{\boldsymbol{e}\alpha}_{\text{context}} + \underbrace{\sum_s \boldsymbol{g}_s\beta_s}_{\text{set}} + \underbrace{\sum_s (\boldsymbol{g}_s \odot \boldsymbol{e})\,\gamma_i}_{\text{set GxC}} + \underbrace{\boldsymbol{\psi}}_{\text{noise}}, \tag{23}$$

with the assumption that the interaction effect for variant i is proportional to its marginal effect (i.e., $\gamma_i = \theta\beta_i$). The presence of GxC interactions can be assessed by considering the one dof test $\theta \neq 0$. For analysis of binary phenotypes, Jiao et al [15] proposed an alternative strategy to re-weight the interaction effects of the variants in the set based on the correlation between the set genotypes and the context variable.

An alternative strategy to aggregate GxC effects across multiple variants is to use a random effect model. Different random-effect models for interaction set test have been proposed [10, 11, 9, 12], all of which build on closely related statistical models. In the following, we describe the Gene-Environment Set Association Test (GESAT) [10], a representative interaction set test that we consider as a comparison partner. The GESAT model is similar to the model in Eq. (23); however, the presence of an interaction is assessed by modelling $\boldsymbol{\gamma}$ as a random effect, $\boldsymbol{\gamma} \sim \mathcal{N}(\boldsymbol{0}, \tau\boldsymbol{I}_R)$, and testing $\tau \neq 0$. This is done by using a score test, similar to [16]. Score tests are attractive, as they do not require to explicitly fit the alternative model. When considering sets with a large numbers of variants, the number of fixed effects in the null model is also large, which may lead to overfitting. To overcome this issue, Lin et al have considered ridge regression to fit the null model [17]. The score test statistics is

$$\boldsymbol{Q} = (\boldsymbol{y} - \hat{\boldsymbol{\mu}})^\top \boldsymbol{S}\boldsymbol{S}^\top (\boldsymbol{y} - \hat{\boldsymbol{\mu}}), \tag{24}$$

where $\boldsymbol{S} = [\boldsymbol{g}_1 \odot \boldsymbol{e}, \dots, \boldsymbol{g}_R \odot \boldsymbol{e}]$ and $\hat{\boldsymbol{\mu}}$ is the optimised mean under the null model (i.e., for $\boldsymbol{\gamma} = 0$). It can be shown that $\boldsymbol{Q}$ follows a mixture of χ^2 distributions with 1 dof [16] and that the coefficients of this mixture are the eigenvalues of the matrix

$$\boldsymbol{T} = \boldsymbol{P}_0^{\frac{1}{2}} \boldsymbol{S}\boldsymbol{S}^\top \boldsymbol{P}_0^{\frac{1}{2}} \in \mathbb{R}^{N \times N}, \tag{25}$$

where $\boldsymbol{P}_0 = \hat{\sigma}_n^2 \boldsymbol{I}_N - \hat{\sigma}_n^2 \tilde{\boldsymbol{F}} \left(\tilde{\boldsymbol{F}}^\top \tilde{\boldsymbol{F}}\right)^{-1} \tilde{\boldsymbol{F}}^\top$ and $\hat{\sigma}_n^2$ is the maximum likelihood estimate of the noise variance under the null and $\tilde{\boldsymbol{F}} = [\boldsymbol{F}, \boldsymbol{e}, \boldsymbol{g}_1, \dots, \boldsymbol{g}_R] \in \mathbb{R}^{N \times (K+R+1)}$. P values can be calculated from the score

test statistics under the assumption that the asymptotic distribution is valid, typically using the Davies method [18]. Computation of the eigenvalue decomposition of $\boldsymbol{T}$ has complexity $O(N^3)$. However, if the number of variants is lower than the number of samples ($R < N$) the non-zero eigenvalues of $\boldsymbol{T}$ can be computed as the eigenvalues of $\boldsymbol{S}^\top \boldsymbol{P}_0 \boldsymbol{S}$, whose computation and eigenvalue decomposition requires $O(NR^2)$ and $O(R^3)$ respectively. For details on score-based methods we refer to [16], [19] and [20].

Building on a multi-trait framework, iSet extends existing interaction set tests in multiple aspects: (i) it enables interaction set test in the analysis of data with either complete or stratified designs, (ii) it explicitly model noise heterogeneity across the different contexts, (iii) it allows for testing different classes of GxC effects, (iv) it enables estimation of variance components. On the other hand, some of the methods mentioned here have features that are not currently available in iSet. Specifically, the model is not designed for rare variant association tests and does not explicitly support case/control phenotypes and continuous environments [11, 21, 12].

2. Extension to stratified designs

In this section, we describe how the iSet model can be used to analyze of stratified samples and compare it to existing set test implementations. We start by extending Eq. (1) to the case of incomplete phenotype designs in Section 2.1 and subsequently focus on the specific design encountered in analysis of stratified samples in Section 2.2. In Section 2.3 we present the efficient implementation of iSet in this data design while in Section 2.4 we present the single-variant version of the same approach, which generalizes the model presented in [6].

2.1. The general case

Let $N_{\text{obs}} \leq NC$ denote the number of phenotype observations and let $\eta(i)$ and $\epsilon(i)$ denote indicator functions that return the individual and the context indices for sample i, respectively. Indicating with $\boldsymbol{Y}$ the $N \times C$ phenotype matrix, containing $N_{\text{miss}} = NC - N_{\text{obs}}$ missing values, and with $\tilde{\boldsymbol{y}}_i = \boldsymbol{Y}_{\eta(i),\epsilon(i)}$ the fully observed phenotype vector, the model in Eq. (1) can be generalized as follows

$$\tilde{\boldsymbol{y}} \sim \mathcal{N}\left(\underbrace{\left(\tilde{\boldsymbol{A}} \mathbin{\widehat{\odot}} \tilde{\boldsymbol{F}}\right)\boldsymbol{b}}_{\text{fixed effect covariates}}, \underbrace{\left(\tilde{\boldsymbol{C}}_s^{1/2} \mathbin{\widehat{\odot}} \tilde{\boldsymbol{G}}\right)\left(\tilde{\boldsymbol{C}}_s^{1/2} \mathbin{\widehat{\odot}} \tilde{\boldsymbol{G}}\right)^T}_{\text{set component}} + \underbrace{\tilde{\boldsymbol{C}}_g \odot \tilde{\boldsymbol{R}}}_{\text{relatedness}} + \underbrace{\tilde{\boldsymbol{C}}_n \odot \tilde{\boldsymbol{N}}}_{\text{noise}}\right) \tag{26}$$

where we have introduced "extended" fixed effects, trait and sample covariances

$$\begin{aligned}
\tilde{\boldsymbol{A}}_{i,:} &= \boldsymbol{I}_{E\ \epsilon(i),:} && \forall i \in \{1, ..., N_{\text{obs}}\} \\
\tilde{\boldsymbol{F}}_{i,:} &= \boldsymbol{F}_{\eta(i),:} && \forall i \in \{1, ..., N_{\text{obs}}\} \\
\tilde{\boldsymbol{C}}_s^{1/2}{}_{i,:} &= \boldsymbol{C}_s^{1/2}{}_{\epsilon(i),:} && \forall i \in \{1, ..., N_{\text{obs}}\} \\
\tilde{\boldsymbol{C}}_{g\ i,j} &= \left(\boldsymbol{C}_g^{1/2}\right)_{\epsilon(i),\epsilon(j)} && \forall i,j \in \{1, ..., N_{\text{obs}}\} \\
\tilde{\boldsymbol{C}}_{n\ i,j} &= \boldsymbol{C}_n^{1/2}{}_{\epsilon(i),\epsilon(j)} && \forall i,j \in \{1, ..., N_{\text{obs}}\} \\
\tilde{\boldsymbol{G}}_{i,:} &= \boldsymbol{G}_{\eta(i),:} && \forall i \in \{1, ..., N_{\text{obs}}\} \\
\tilde{\boldsymbol{R}}_{i,j} &= \boldsymbol{R}_{\eta(i),\eta(j)} && \forall i,j \in \{1, ..., N_{\text{obs}}\} \\
\tilde{\boldsymbol{N}}_{i,j} &= \boldsymbol{I}_{\eta(i),\eta(j)} && \forall i,j \in \{1, ..., N_{\text{obs}}\}
\end{aligned}$$

and the operation $\widehat{\odot} : (\mathbb{R}^{n\times m}, \mathbb{R}^{n\times l}) \to \mathbb{R}^{n\times ml}$, defined as follows

$$\boldsymbol{A} \mathbin{\widehat{\odot}} \boldsymbol{B} = \left(\boldsymbol{A} \otimes \boldsymbol{1}^T_{\text{cols}(\boldsymbol{B})}\right) \odot \left(\boldsymbol{1}^T_{\text{cols}(\boldsymbol{A})} \otimes \boldsymbol{B}\right). \tag{27}$$

Note that $\widehat{\odot}$ between $\boldsymbol{A} \in \mathbb{R}^{n\times m}$ and an $\boldsymbol{B} \in \mathbb{R}^{n\times l}$ gives the $n \times ml$ matrix whose columns corresponds the pair-wise Hadamart product of the columns $\boldsymbol{A}$ and $\boldsymbol{B}$.

2.2. The incomplete design for stratification analysis

In the case where the phenotype data for each sample are available only in one of the the contexts, we have $\eta(i) = i$ and the individual noise matrix $\tilde{\boldsymbol{N}}$ reduces to the identity matrix. As consequence, the noise covariance term has diagonal form[2]. Without loss of generality, we can re-order the observations such that the first N_1 of them correspond to context 1 and the last $N - N_1$ correspond to context 2

$$\epsilon(i) = \begin{cases} 1 & i < N_1 \\ 2 & \text{otherwise} \end{cases} \tag{28}$$

Equation (26) becomes:

$$\tilde{\boldsymbol{y}} \sim \mathcal{N}\left(\underbrace{\left(\tilde{\boldsymbol{A}} \mathbin{\widehat{\odot}} \boldsymbol{F}\right)\boldsymbol{b}}_{\text{fixed effect covariates}}, \underbrace{\left(\tilde{\boldsymbol{C}}_s^{1/2} \mathbin{\widehat{\odot}} \boldsymbol{G}\right)\left(\tilde{\boldsymbol{C}}_s^{1/2} \mathbin{\widehat{\odot}} \boldsymbol{G}\right)^T}_{\text{set component}} + \underbrace{\tilde{\boldsymbol{C}}_g \odot \boldsymbol{R}}_{\text{relatedness}} + \underbrace{\boldsymbol{D}}_{\text{noise}}\right) \tag{29}$$

where

$$\boldsymbol{D} = \text{diag}(\underbrace{\sigma_1^2, \dots, \sigma_1^2}_{N_1}, \underbrace{\sigma_2^2, \dots, \sigma_2^2}_{N-N_1}) \tag{30}$$

and σ_1^2 and σ_2^2 indicate the noise variance in context 1 and 2 respectively.

2.3. iSet for stratification analysis in unrelated individual

In analysis of unrelated individuals genetic relatedness can be modeled as fixed effect using the top principal components of the realized relationship matrix while the relatedness covariance term in Eq. (31) can be dropped

$$\tilde{\boldsymbol{y}} \sim \mathcal{N}\left(\underbrace{\boldsymbol{X}\boldsymbol{b}}_{\text{fixed effect covariates}}, \underbrace{\boldsymbol{W}_{\boldsymbol{\theta}}\boldsymbol{W}_{\boldsymbol{\theta}}^T}_{\text{set component}} + \underbrace{\boldsymbol{D}_{\boldsymbol{\theta}}}_{\text{noise}}\right) \tag{31}$$

where we introduced $\boldsymbol{X} = \left(\tilde{\boldsymbol{A}} \mathbin{\widehat{\odot}} \boldsymbol{F}\right)$, $\boldsymbol{W}_{\boldsymbol{\theta}} = \tilde{\boldsymbol{C}}_s^{1/2} \mathbin{\widehat{\odot}} \boldsymbol{G}$ and have explicitly indicated dependency on model parameters $\boldsymbol{\theta}$. Similarly to mtSet [2], parameter inference in iSet is done by using a gradient-based parameter optimization (LBFGS [22, 23]), which requires computation of the log marginal likelihood (LML) and its gradients. In the following we discuss implementation details and the fast inference scheme used by iSet for stratification analysis in unrelated individuals.

LML and its gradients The (restricted) LML of the model and its gradients are [24, 25]

$$\mathcal{L}_{\boldsymbol{\theta}} = -\frac{1}{2}\log|\boldsymbol{K}_{\boldsymbol{\theta}}| - \frac{1}{2}\log|\boldsymbol{A}_{\boldsymbol{\theta}}| - \frac{1}{2}\boldsymbol{y}^\top\boldsymbol{K}_{\boldsymbol{\theta}}^{-1}\boldsymbol{y} + \frac{1}{2}\boldsymbol{y}^\top\boldsymbol{K}_{\boldsymbol{\theta}}^{-1}\boldsymbol{X}\boldsymbol{b}_{\boldsymbol{\theta}} \tag{32}$$

$$\begin{aligned}\frac{\partial\mathcal{L}_{\boldsymbol{\theta}}}{\partial\boldsymbol{\theta}_i} &= -\frac{1}{2}\text{tr}\left(\boldsymbol{K}_{\boldsymbol{\theta}}^{-1}\frac{\partial\boldsymbol{K}_{\boldsymbol{\theta}}}{\partial\boldsymbol{\theta}_i}\right) - \frac{1}{2}\text{tr}\left(\boldsymbol{A}_{\boldsymbol{\theta}}^{-1}\frac{\partial\boldsymbol{A}_{\boldsymbol{\theta}}}{\partial\boldsymbol{\theta}_i}\right) + \frac{1}{2}\boldsymbol{y}^\top\boldsymbol{K}_{\boldsymbol{\theta}}^{-1}\frac{\partial\boldsymbol{K}_{\boldsymbol{\theta}}}{\partial\boldsymbol{\theta}_i}\boldsymbol{K}_{\boldsymbol{\theta}}^{-1}\boldsymbol{y} \\ &\quad -\boldsymbol{y}^\top\boldsymbol{K}_{\boldsymbol{\theta}}^{-1}\frac{\partial\boldsymbol{K}_{\boldsymbol{\theta}}}{\partial\boldsymbol{\theta}_i}\boldsymbol{K}_{\boldsymbol{\theta}}^{-1}\boldsymbol{X}\boldsymbol{b}_{\boldsymbol{\theta}} - \frac{1}{2}\boldsymbol{b}_{\boldsymbol{\theta}}^\top\frac{\partial\boldsymbol{A}_{\boldsymbol{\theta}}}{\partial\boldsymbol{\theta}_i}\boldsymbol{b}_{\boldsymbol{\theta}}\end{aligned} \tag{33}$$

[2] Intuitively, estimation of noise correlation is not possible as there are no repeated measures (across contexts) for the same individual.

where

$$\begin{aligned} \boldsymbol{K}_{\boldsymbol{\theta}} &= \boldsymbol{W}_{\boldsymbol{\theta}}\boldsymbol{W}_{\boldsymbol{\theta}}^T + \boldsymbol{D}_{\boldsymbol{\theta}} & (34)\\ \boldsymbol{A}_{\boldsymbol{\theta}} &= \boldsymbol{X}^\top \boldsymbol{K}_{\boldsymbol{\theta}}^{-1}\boldsymbol{X} & (35)\\ \boldsymbol{b}_{\boldsymbol{\theta}} &= \boldsymbol{A}_{\boldsymbol{\theta}}^{-1}\boldsymbol{X}^\top \boldsymbol{K}_{\boldsymbol{\theta}}^{-1}\boldsymbol{y}, & (36) \end{aligned}$$

$$\begin{aligned} \frac{\partial \boldsymbol{A}_{\boldsymbol{\theta}}}{\partial \boldsymbol{\theta}_i} &= -\boldsymbol{X}^\top \boldsymbol{K}_{\boldsymbol{\theta}}^{-1}\frac{\partial \boldsymbol{K}_{\boldsymbol{\theta}}}{\partial \boldsymbol{\theta}_i}\boldsymbol{K}_{\boldsymbol{\theta}}^{-1}\boldsymbol{X}, & (37)\\ \frac{\partial \boldsymbol{K}}{\partial \boldsymbol{\theta}_i} &= \begin{cases} \boldsymbol{W}\frac{\partial \boldsymbol{W}}{\partial \boldsymbol{\theta}_i}^T + \frac{\partial \boldsymbol{W}}{\partial \boldsymbol{\theta}_i}\boldsymbol{W}^T & \text{if } i \leq \frac{C(C+1)}{2} \\ \frac{\partial \boldsymbol{D}}{\partial \boldsymbol{\theta}_i} & \text{otherwise} \end{cases}, & (38) \end{aligned}$$

$$\begin{aligned} \boldsymbol{K}^{-1} &= \boldsymbol{D}^{-1} - \boldsymbol{D}^{-1}\boldsymbol{W}\boldsymbol{H}^{-1}\boldsymbol{W}^T\boldsymbol{D}^{-1}, & (39)\\ \boldsymbol{H} &= \boldsymbol{I} + \boldsymbol{W}^T\boldsymbol{D}^{-1}\boldsymbol{W} & (40) \end{aligned}$$

As shown in more detail in the next paragraph, evaluation of a LML and its gradients has complexity $O(NR^2 + NK^2 + R^3 + K^3)$.

Computational complexity of all terms We denote with $\boxed{\cdots}$ the formulae parts which have been pre-computed at a given stage.

- $\boldsymbol{D}^{-1}\boldsymbol{W}$

  $O(NR)$

- $\boldsymbol{H} = \boldsymbol{I} + \boldsymbol{W}^T \boxed{\boldsymbol{D}^{-1}\boldsymbol{W}}$

  $O(NR^2)$

- cholesky $(\boldsymbol{H})$

  $O(R^3)$

- $\boldsymbol{K}^{-1}\boldsymbol{y} = \boldsymbol{D}^{-1} - \boldsymbol{D}^{-1}\boldsymbol{W}\boldsymbol{H}^{-1}\boldsymbol{W}^T\boldsymbol{D}^{-1}\boldsymbol{y}$

  $O(N + NR)$

- $\boldsymbol{K}^{-1}\boldsymbol{X} = \boldsymbol{D}^{-1} - \boldsymbol{D}^{-1}\boldsymbol{W}\boldsymbol{H}^{-1}\boldsymbol{W}^T\boldsymbol{D}^{-1}\boldsymbol{X}$

  $O(NK + NRK)$

- $\boldsymbol{A} = \boldsymbol{X}^T \boxed{\boldsymbol{K}^{-1}\boldsymbol{X}}$

  $O(NK^2)$

- cholesky $(\boldsymbol{A})$

$$O(K^3)$$

- $\boldsymbol{b} = \boldsymbol{A}^{-1}\boldsymbol{X}^T \boxed{\boldsymbol{K}^{-1}\boldsymbol{y}}$

$$O(NK + K^2)$$

- $\boxed{\boldsymbol{K}^{-1}\boldsymbol{X}}\boldsymbol{b}$

$$O(NK)$$

- $\log\det \boldsymbol{K} = \log\det(\boldsymbol{H}) - \log\det\left(\boldsymbol{D}^{-1}\right)$

$$O(N + R)$$

- $\frac{\partial \boldsymbol{K}}{\partial \boldsymbol{\theta}_i}\boxed{\boldsymbol{K}^{-1}\boldsymbol{y}} = \begin{cases} \boldsymbol{W}\frac{\partial \boldsymbol{W}}{\partial \boldsymbol{\theta}_i}^T \boxed{\boldsymbol{K}^{-1}\boldsymbol{y}} + \frac{\partial \boldsymbol{W}}{\partial \boldsymbol{\theta}_i}\boldsymbol{W}^T \boxed{\boldsymbol{K}^{-1}\boldsymbol{y}} & \text{if } i \leq \frac{E(E+1)}{2} \\ \frac{\partial \boldsymbol{D}}{\partial \boldsymbol{\theta}_i}\boxed{\boldsymbol{K}^{-1}\boldsymbol{y}} & \text{otherwise} \end{cases}$

$$O(NR + N)$$

- $\frac{\partial \boldsymbol{K}}{\partial \boldsymbol{\theta}_i}\boxed{\boldsymbol{K}^{-1}\boldsymbol{X}} = \begin{cases} \boldsymbol{W}\frac{\partial \boldsymbol{W}}{\partial \boldsymbol{\theta}_i}^T \boxed{\boldsymbol{K}^{-1}\boldsymbol{X}} + \frac{\partial \boldsymbol{W}}{\partial \boldsymbol{\theta}_i}\boldsymbol{W}^T \boxed{\boldsymbol{K}^{-1}\boldsymbol{X}} & \text{if } i \leq \frac{E(E+1)}{2} \\ \frac{\partial \boldsymbol{D}}{\partial \boldsymbol{\theta}_i}\boxed{\boldsymbol{K}^{-1}\boldsymbol{X}} & \text{otherwise} \end{cases}$

$$O(NRK + NK)$$

- $\frac{\partial \boldsymbol{A}_{\boldsymbol{\theta}}}{\partial \boldsymbol{\theta}_i} = -\boxed{\boldsymbol{X}^\top \boldsymbol{K}_{\boldsymbol{\theta}}^{-1}}\boxed{\frac{\partial \boldsymbol{K}_{\boldsymbol{\theta}}}{\partial \boldsymbol{\theta}_i}\boldsymbol{K}_{\boldsymbol{\theta}}^{-1}\boldsymbol{X}}$

$$O(NK^2)$$

- $\operatorname{tr}\left(\boldsymbol{K}^{-1}\frac{\partial \boldsymbol{K}}{\partial \boldsymbol{\theta}_i}\right) = \sum_{ii} \boldsymbol{D}_{ii}^{-1}\left(\frac{\partial \boldsymbol{K}}{\partial \boldsymbol{\theta}_i}\right)_{ii} - \sum_{ij} \boldsymbol{H}_{ij}^{-1}\left(\boldsymbol{W}^T\boldsymbol{D}^{-1}\frac{\partial \boldsymbol{K}}{\partial \boldsymbol{\theta}_i}\boldsymbol{D}^{-1}\boldsymbol{W}\right)_{ij}$

$$= \begin{cases} 2\sum_{ii} \boldsymbol{D}_{ii}^{-1}\left(\frac{\partial \boldsymbol{W}}{\partial \boldsymbol{\theta}_i}\boldsymbol{W}^T\right)_{ii} - \sum_{ij} \boldsymbol{H}_{ij}^{-1}\left(\boxed{\boldsymbol{D}^{-1}\boldsymbol{W}}^T \frac{\partial \boldsymbol{W}}{\partial \boldsymbol{\theta}_i}\boxed{\boldsymbol{W}^T\boldsymbol{D}^{-1}\boldsymbol{W}} + (\text{transp})\right)_{ij} \\ \sum_{ii} \boldsymbol{D}_{ii}^{-1}\left(\frac{\partial \boldsymbol{D}}{\partial \boldsymbol{\theta}_i}\right)_{ii} - \sum_{ij} \boldsymbol{H}_{ij}^{-1}\left(\boxed{\boldsymbol{D}^{-1}\boldsymbol{W}}^T \frac{\partial \boldsymbol{D}}{\partial \boldsymbol{\theta}_i}\boxed{\boldsymbol{D}^{-1}\boldsymbol{W}}\right)_{ij} \end{cases}$$

$$O(NR + N + NR^2 + R^3 + R^2)$$

2.4. Single-variant models for stratified designs

We extended the single-variant multi-trait linear mixed model in [6] so that it is applicable for analysis of stratified samples. The model is similar to Eq. eq:LMMexcl with a fixed effect from the SNP being tested and no effect from the genetic region:

$$\tilde{\boldsymbol{y}} \sim \mathcal{N}\left(\underbrace{\boldsymbol{X}\boldsymbol{b}}_{\text{fixed effect covariates}} + \underbrace{\left(\left(\boldsymbol{1}_C^T \otimes \boldsymbol{g}\right) \odot \boldsymbol{E}\right)\boldsymbol{w}^T}_{\text{variant effect}}, \underbrace{\boldsymbol{D}_{\boldsymbol{\theta}}}_{\text{noise}}\right) \tag{41}$$

where $\boldsymbol{g}$ is the genotype of the SNP, $\boldsymbol{E} \in \mathbb{R}^{N \times C}$ a context indicator[3] and $\boldsymbol{w} \in \mathbb{R}^{C}$ the vector of variant effect sizes across the different C contexts. The is similar to single-variant interaction models but allows different effect sizes of covariates and noise levels across the analyzed contexts. The statistical test for GxC interaction corresponds to $w_1 \neq w_2$. Again, variance components are learnt in the no-association model ($\boldsymbol{w} = \boldsymbol{0}$) and kept constant when fitting the common effect and the full models.

3. Simulation experiments

In this section we present the implementation details of the simulation experiments.

3.1. Genotype data

Following the procedure used in [26, 2] we generated synthetic genotype data based on real genotypes from 1000 Genomes Project individuals of European ancestry [27] (populations: CEU, FIN, GBR, IBS, TSI). Briefly, for each of the new individuals we randomly assign a population of origin (CEU, FIN, GBR, IBS, TSI) and 10 ancestors from that population. Then we split the genome in blocks of 1,000 variants and synthesize his genome as mosaic of blocks of 1,000 SNPs and then randomly assigned each block to one of the ancestors. This strategy generates genotype data with unrelated individuals and population structure (see discussion in [2]).

3.2. Phenotype simulation strategy

Following [2], we generated trait measurements in two contexts as the sum of the contribution from a randomly-selected causal region of 30kb ($\boldsymbol{S}$), polygenic background effect ($\boldsymbol{G}$), effects from $K = 10$ unobserved covariates ($\boldsymbol{H}$) and iid noise ($\boldsymbol{\Psi}$):

$$\boldsymbol{Y} = \boldsymbol{S} + \boldsymbol{G} + \boldsymbol{H} + \boldsymbol{\Psi}. \tag{42}$$

Genetic effects from the causal region were simulated to generate persistent, rescaling-GxC or general-GxC effects (rescaling-GxC effects + heterogeneity-GxC effects).

- **Persistent and rescaling-GxC effects.** S_c causal variants were randomly selected from the region. Denoting with $\boldsymbol{G} \in \mathbb{R}^{N \times S_c}$ the standardised genotypes of the selected causal variants, the local polygenic effect was simulated as

$$\boldsymbol{S} = \boldsymbol{G}\boldsymbol{b}\,[1, \eta]\,, \quad \text{where } \boldsymbol{b} \overset{\text{iid}}{\sim} \{-1, +1\}\,, \tag{43}$$

  where $\boldsymbol{b} \in \mathbb{R}^{S_c}$ is the effect size of the causal variants and η is the proportionality factor of the effect sizes across the two contexts. Note that η can be positive (positive rescaling), negative (negative rescaling) or zero (i.e. the polygenic effect is specific to the first context). Additionally, $\eta = 1$ corresponds to the case of persistent effects.

- **General-GxC.** When simulating general-GxC, we included scenarios with different causal variants between the two contexts. To do so, we independently sampled s_c causal variants in each of the two contexts, resulting in a total of $S_c = 2s_c$ variants. In each context, the causal variants were selected such that all pairwise squared Pearson correlations were lower than 0.4. This was done by rejecting sampled configurations that did not satisfy this condition. Denoting with $\boldsymbol{G}_1 \in \mathbb{R}^{N \times s_C}$ and $\boldsymbol{G}_2 \in \mathbb{R}^{N \times s_C}$ the standardised genotypes of the casual variants in the two contexts and with

[3] $\boldsymbol{E}_{ic}$ is one if sample i is observed in context c, 0 otherwise.

$\boldsymbol{b}_1 \in \mathbb{R}^{s_C}$ and $\boldsymbol{b}_2 \in \mathbb{R}^{s_C}$ the respective vectors of their effect sizes, the local polygenetic effect was generated as

$$\boldsymbol{S} = \begin{bmatrix}\boldsymbol{G}_1 & \boldsymbol{G}_2\end{bmatrix}\begin{bmatrix}\boldsymbol{b}_1 & \boldsymbol{0}_{s_c \times 1} \\ \boldsymbol{0}_{s_c \times 1} & \boldsymbol{b}_2\end{bmatrix}, \quad \text{where } \boldsymbol{b}_1, \boldsymbol{b}_2 \overset{\text{iid}}{\sim} \{-1, +1\}\,. \tag{44}$$

The extent of heterogeneity-GxC was controlled by additionally controlling the correlation of the polygenetic effects between the two contexts. In particular, given a certain target correlation range $[\rho_m, \rho_M]$, we only considered realisations for which

$$\rho_m < \operatorname{corr}(\boldsymbol{S}_{:,1}, \boldsymbol{S}_{:,2}) < \rho_M, \tag{45}$$

by rejecting realisations that did not satisfy this condition.

The genetic contributions from the regions were scaled so that $v_{\text{s}} = \text{var}\left[\text{vec}(\boldsymbol{R})\right] = 2\%$. When considering the general-GxC case, to limit the extent of rescaling-GxC, we rescaled each column of $\boldsymbol{R}$ to have variance 2%.

The effects from population structure and unobserved covariates were generated as the sum of a shared and an independent component across contexts.

$$\begin{aligned}
\boldsymbol{G} &= \boldsymbol{G}^{(\text{s})} + \boldsymbol{G}^{(\text{i})} && (46)\\
\boldsymbol{H} &= \boldsymbol{H}^{(\text{s})} + \boldsymbol{H}^{(\text{i})} && (47)\\
\boldsymbol{G}^{(\text{s})} &\sim \text{MVN}\left(\boldsymbol{0}, \boldsymbol{R}, \boldsymbol{a}_{\text{G}}\boldsymbol{a}_{\text{G}}^{\top}\right) && (48)\\
\boldsymbol{G}^{(\text{i})} &\sim \text{MVN}\left(\boldsymbol{0}, \boldsymbol{R}, \text{diag}(\boldsymbol{c}_{\text{G}}^2)\right) && (49)\\
\boldsymbol{H}^{(\text{s})} &\sim \text{MVN}\left(\boldsymbol{M}\boldsymbol{M}^{\top}, \boldsymbol{a}_{\text{H}}\boldsymbol{a}_{\text{H}}^{\top}\right) && (50)\\
\boldsymbol{H}^{(\text{i})} &\sim \text{MVN}\left(\boldsymbol{M}\boldsymbol{M}^{\top}, \text{diag}(\boldsymbol{c}_{\text{H}}^2)\right) && (51)
\end{aligned}$$

where $\boldsymbol{M} \in \mathbb{R}^{N \times K}$ is the design matrix of the hidden confounders, $\boldsymbol{R}$ denotes the global realised relatedness matrix and

$$\begin{aligned}
\boldsymbol{a}_{\text{G}} = \sqrt{\boldsymbol{\alpha}_{\text{G}}}, \quad \boldsymbol{c}_{\text{G}} = \sqrt{\boldsymbol{\gamma}_{\text{G}}}, \quad \boldsymbol{a}_{\text{H}} = \sqrt{\boldsymbol{\alpha}_{\text{H}}}, \quad \boldsymbol{c}_{\text{H}} = \sqrt{\boldsymbol{\gamma}_{\text{H}}} && (52)\\
\boldsymbol{\alpha}_{\text{G}}, \boldsymbol{\gamma}_{\text{G}}, \boldsymbol{\alpha}_{\text{H}}, \boldsymbol{\gamma}_{\text{H}} \sim \text{Uniform}(0,1) && (53)\\
\boldsymbol{M}_{i,j} \sim \mathcal{N}(0,1) \quad i = 1,\ldots,N, \quad k = 1,\ldots,K. && (54)
\end{aligned}$$

Denoting with α the fraction of shared signal and with β the fraction of residual variance that is non-iid, the different contributions were rescaled such that

$$\begin{aligned}
\text{var}\left[\text{vec}(\boldsymbol{G}^{(\text{s})})\right] &= \alpha v_{\text{bg}} && (55)\\
\text{var}\left[\text{vec}(\boldsymbol{G}^{(\text{i})})\right] &= (1-\alpha) v_{\text{bg}} && (56)\\
\text{var}\left[\text{vec}(\boldsymbol{H}^{(\text{s})})\right] &= \alpha\beta(1 - v_{\text{bg}} - v_{\text{s}}) && (57)\\
\text{var}\left[\text{vec}(\boldsymbol{H}^{(\text{s})})\right] &= (1-\alpha)\beta(1 - v_{\text{bg}} - v_{\text{s}}) && (58)\\
\text{var}\left[\text{vec}(\boldsymbol{\Psi})\right] &= (1-\beta)(1 - v_{\text{bg}} - v_{\text{s}}) && (59)
\end{aligned}$$

Unless specified otherwise, we considered the default values $v_{\text{s}} = 2\%$, $v_{\text{bg}} = 40\%$, $\alpha = 0.6$ and $\beta = 0.5$. When simulating scenarios with rescaling-GxC we varied the number of causal variants (S_c) and proportionality factor of the effects in the two contexts (η, which quantifies the extent of rescaling). When simulating general-GxC (rescaling-GxC + heterogeneity-GxC) we varied the number of causal variants (S_c) and the range of correlations between the genetic effects in the two contexts ($[\rho_m, \rho_M]$), thereby varying the extent of heterogeneity-GxC. A summary of the parameter values used in simulations is provided in **Supplementary Table 2**.

A. Kronecker product and operations

A.1. Definition of the Kronecker product

$\boldsymbol{A} \otimes \boldsymbol{B}$ between the two arbitrary two matrices $\boldsymbol{A} \in \mathbb{R}^{M\times N}, \boldsymbol{B} \in \mathbb{R}^{Q\times R}$ is defined as

$$\boldsymbol{A} \otimes \boldsymbol{B} = \begin{pmatrix} A_{11}\boldsymbol{B} & \dots & A_{1N}\boldsymbol{B} \\ \vdots & \ddots & \vdots \\ A_{M1}\boldsymbol{B} & \dots & A_{MN}\boldsymbol{B} \end{pmatrix} \tag{60}$$

and has the dimension $MQ \times NR$. The vec operator concatenates the columns an arbitrary matrix $\boldsymbol{A}$ into a vector of length $M \cdot N$.

A.2. Matrix variate normals models as normal distribution

The matrix variate normal distribution

$$\boldsymbol{X} \sim MVN(\boldsymbol{M}, \boldsymbol{C}, \boldsymbol{R}) \tag{61}$$

is equivalent to the multivariate normal distribution

$$\text{vec}(\boldsymbol{X}) \sim \mathcal{N}\left(\text{vec}(\boldsymbol{X})\,|\text{vec}(\boldsymbol{M}), \boldsymbol{C} \otimes \boldsymbol{R}\right). \tag{62}$$

References

[1] Lee, S. H., Goddard, M. E., Visscher, P. M. & van der Werf, J. H. Using the realized relationship matrix to disentangle confounding factors for the estimation of genetic variance components of complex traits. *Genet Sel Evol* **42**, 22 (2010).

[2] Casale, F. P., Rakitsch, B., Lippert, C. & Stegle, O. Efficient set tests for the genetic analysis of correlated traits. *Nat Methods* **12**, 755–8 (2015).

[3] Bůžková, P., Lumley, T. & Rice, K. Permutation and parametric bootstrap tests for gene-gene and gene-environment interactions. *Ann Hum Genet* **75**, 36–45 (2011).

[4] Searle, S. R. Matrix algebra useful for statistics (wiley series in probability and statistics) (1982).

[5] Kostem, E. & Eskin, E. Improving the accuracy and efficiency of partitioning heritability into the contributions of genomic regions. *Am. J. Hum. Genet.* **92**, 558–64 (2013).

[6] Korte, A. *et al.* A mixed-model approach for genome-wide association studies of correlated traits in structured populations. *Nature genetics* **44**, 1066–1071 (2012).

[7] Furlotte, N. A. & Eskin, E. Efficient multiple-trait association and estimation of genetic correlation using the matrix-variate linear mixed model. *Genetics* **200**, 59–68 (2015).

[8] Chatterjee, N., Kalaylioglu, Z., Moslehi, R., Peters, U. & Wacholder, S. Powerful multilocus tests of genetic association in the presence of gene-gene and gene-environment interactions. *Am J Hum Genet* **79**, 1002–16 (2006).

[9] Tzeng, J.-Y. *et al.* Studying gene and gene-environment effects of uncommon and common variants on continuous traits: a marker-set approach using gene-trait similarity regression. *Am J Hum Genet* **89**, 277–88 (2011).

[10] Lin, X., Lee, S., Christiani, D. C. & Lin, X. Test for interactions between a genetic marker set and environment in generalized linear models. *Biostatistics* **14**, 667–81 (2013).

[11] Lin, X. *et al.* Test for rare variants by environment interactions in sequencing association studies. *Biometrics* **72**, 156–64 (2016).

[12] Zhao, G., Marceau, R., Zhang, D. & Tzeng, J.-Y. Assessing gene-environment interactions for common and rare variants with binary traits using gene-trait similarity regression. *Genetics* **199**, 695–710 (2015).

[13] Zhou, X. & Stephens, M. Efficient multivariate linear mixed model algorithms for genome-wide association studies. *Nat Methods* **11**, 407–9 (2014).

[14] Lippert, C., Casale, F. P., Rakitsch, B. & Stegle, O. Limix: genetic analysis of multiple traits. *bioRxiv* 003905 (2014).

[15] Jiao, S. *et al.* Sberia: set-based gene-environment interaction test for rare and common variants in complex diseases. *Genet Epidemiol* **37**, 452–64 (2013).

[16] Wu, M. C. *et al.* Rare-variant association testing for sequencing data with the sequence kernel association test. *Am. J. Hum. Genet.* **89**, 82–93 (2011).

[17] Hoerl, A. E. & Kennard, R. W. Ridge regression: Biased estimation for nonorthogonal problems. *Technometrics* **12**, 55–67 (1970).

[18] Davies, R. B. Algorithm as 155: The distribution of a linear combination of χ 2 random variables. *Journal of the Royal Statistical Society. Series C (Applied Statistics)* **29**, 323–333 (1980).

[19] Lee, S. *et al.* Optimal unified approach for rare-variant association testing with application to small-sample case-control whole-exome sequencing studies. *Am. J. Hum. Genet.* **91**, 224–37 (2012).

[20] Lippert, C. *et al.* Greater power and computational efficiency for kernel-based association testing of sets of genetic variants. *Bioinformatics* **30**, 3206–14 (2014).

[21] Broadaway, K. A. *et al.* Kernel approach for modeling interaction effects in genetic association studies of complex quantitative traits. *Genet Epidemiol* **39**, 366–75 (2015).

[22] Liu, D. C. & Nocedal, J. On the limited memory bfgs method for large scale optimization. *Mathematical programming* **45**, 503–528 (1989).

[23] Zhu, C., Byrd, R. H., Lu, P. & Nocedal, J. Algorithm 778: L-bfgs-b: Fortran subroutines for large-scale bound-constrained optimization. *ACM Transactions on Mathematical Software (TOMS)* **23**, 550–560 (1997).

[24] Harville, D. A. Bayesian inference for variance components using only error contrasts. *Biometrika* **61**, 383–385 (1974).

[25] LaMotte, L. R. A direct derivation of the reml likelihood function. *Statistical Papers* **48**, 321–327 (2007).

[26] Loh, P.-R. *et al.* Efficient bayesian mixed-model analysis increases association power in large cohorts. *Nat Genet* **47**, 284–90 (2015).

[27] 1000 Genomes Project Consortium et al. An integrated map of genetic variation from 1,092 human genomes. *Nature* **491**, 56–65 (2012).
