# Supplementary material for: Joint genetic analysis using variant sets reveals polygenic gene-context interactions
Source: PLoS Genet. 2017 Apr 20;13(4):e1006693. doi: 10.1371/journal.pgen.1006693 (PMC5398484; doi:10.1371/journal.pgen.1006693)
Supplement: S1 Table — Shown are empirical type-1 error estimates for increasingly stringent significance level thresholds. Persistent genetic effects (No GxC effects) were simulated using the standard simulation parameters (a, parameters in S2 Table but without simulating rescaling) and the same setting while considering a single causal variant (b), outlying samples (c) and epistatic interactions between randomly selected pairs of variants (d). These results show that iSet and iSet-het yield P values with controlled type-I error rates under different types of model misspecification. The corresponding QQ plots are shown in Fig 2A (main) and S4 Fig. (PDF) [file pgen.1006693.s002.pdf]

Significance level	iSet	iSet-het
5.00E-02	5.11E-02	5.50E-02
5.00E-03	5.06E-03	5.77E-03
5.00E-04	4.00E-04	6.11E-04
5.00E-05	3.33E-05	3.33E-05

(a) 4 causal variant

Significance level	iSet	iSet-het
5.00E-02	5.17E-02	5.48E-02
5.00E-03	5.31E-03	5.58E-03
5.00E-04	4.22E-04	5.22E-04
5.00E-05	3.33E-05	1.11E-05

(b) 1 causal variant

Significance level	iSet	iSet-het
5.00E-02	5.24E-02	5.51E-02
5.00E-03	5.40E-03	6.14E-03
5.00E-04	4.89E-04	5.11E-04
5.00E-05	5.56E-05	4.44E-05

(c) Outlying samples

Significance level	iSet	iSet-het
5.00E-02	5.38E-02	5.54E-02
5.00E-03	5.33E-03	4.87E-03
5.00E-04	5.11E-04	4.33E-04
5.00E-05	2.22E-05	1.11E-05

(d) GxG interactions
