# Supplementary material for: Joint genetic analysis using variant sets reveals polygenic gene-context interactions
Source: PLoS Genet. 2017 Apr 20;13(4):e1006693. doi: 10.1371/journal.pgen.1006693 (PMC5398484; doi:10.1371/journal.pgen.1006693)
Supplement: S2 Table — Simulated phenotype data were generated as sum of effects from variants in a designated causal region, effects from a relatedness component/population structure, effects from K = 10 unmeasured hidden confounding factors, and iid observation noise (S1 Text). We fixed the variance explained by the region (vr = 2%), and the fraction of shared background signal (α = 0.6), the fraction of residual variance that is explained by the hidden factors (β = 0.5). We considered variable numbers of causal variants and altered the extent of rescaling-GxC and heterogeneity-GxC. (a) The contributions to phenotypic variance of all simulated effects. (b,c) Parameter values considered for simulations of rescaling-GxC and heterogeneity-GxC effects, respectively. Individual parameters in (b,c) were varied one by one, while keeping others at their default values (bold face). When simulating general-GxC, and when varying the number of causal variants, the region effect correlation r was constrained to 0.2 < r < 0.8. See S1 Text for full details. (PDF) [file pgen.1006693.s003.pdf]

Effect	Function of parameters	Variance explained
Region effect	v_r	2%
Shared relatedness effects	αv_{bg}	24%
Independent relatedness effects	$(1-\alpha)v_{bg}$	16%
Shared effects from hidden factors	$\alpha\beta(1-v_r-v_{bg})$	17.4%
Independent effects from hidden factors	$(1-\alpha)\beta(1-v_r-v_{bg})$	11.6%
IID noise	$(1-\beta)(1-v_r-v_{bg})$	29%

(a) Variance explained by the different simulated factors

Parameter										
Number of causal variants	1	2	**4**	8	12	20	-	-	-	-
Proportionality factor of the effect sizes	-0.5	-0.3	-0.2	-0.1	0	0.1	0.2	**0.3**	0.5	0.7

(b) Parameter values for simulated rescaling-GxC effects‘

Parameter										
Number of causal variants (across contexts)	2	**4**	8	12	20	-	-	-	-	-
Region effect correlation (extent of heter-GxC)	-1, -.8	-.8, -.6	-.6, -.4	-.4, -0.2	-.2, 0	**0, .2**	**.2, .4**	**.4, .6**	**6, .8**	.8, 1

(c) Parameter values for general-GxC effects
