# Supplementary material for: Joint genetic analysis using variant sets reveals polygenic gene-context interactions
Source: PLoS Genet. 2017 Apr 20;13(4):e1006693. doi: 10.1371/journal.pgen.1006693 (PMC5398484; doi:10.1371/journal.pgen.1006693)
Supplement: S3 Table — Shown is the relationship between the proportionality factor of the effect sizes in the two simulated contexts (η, x-axis in Fig 2C), the corresponding fold change of the effect sizes, the fold change of the variance explained by the region and the relative directionality of the genetic signals. For −1 < η < 1, the absolute fold change increases for small absolute values of η and tends to infinity in the limit η → 0. The range η < 0 corresponds to genetic effect with opposite effects. The setting η = 0 corresponds to a local genetic effect that is specific to one context (no effect in the second context). Finally, η = 1 corresponds to no interaction (the fold change is zero and the direction of genetic effects is the same). (PDF) [file pgen.1006693.s004.pdf]

Proportionality factor of the effect sizes (η)	Absolute fold change of the effect sizes	Absolute fold change of the variance explained	Relative direction of the genetic signals
-1	1	1	opposite
-0.7	1.43	2.04	opposite
-0.5	2	4	opposite
-0.3	3.33	11.11	opposite
-0.2	5	25	opposite
-0.1	10	100	opposite
0	inf	inf	N/A
0.1	10	100	same
0.2	5	25	same
0.3	3.33	11.11	same
0.5	2	4	same
0.7	1.43	2.04	same
1	1	1	same
