# Supplementary material for: Joint genetic analysis using variant sets reveals polygenic gene-context interactions
Source: PLoS Genet. 2017 Apr 20;13(4):e1006693. doi: 10.1371/journal.pgen.1006693 (PMC5398484; doi:10.1371/journal.pgen.1006693)
Supplement: S5 Table — Breakdown of probe/stimulus pairs with shared lead variants, stratified by concordance of the effect direction (opposite-direction versus same-direction eQTLs) and significance of the heterogeneity-GxC test (heter vs No heter) in naive/IFN (a), naive/LPS2 (b) and naive/LPS24 (c). (PDF) [file pgen.1006693.s006.pdf]

	Opposite Direction	Same Direction	Fold Change	P-value
heter / No heter	3 / 12	81 / 1444	4.5	0.04

(a) IFN

	Opposite Direction	Same Direction	Fold Change	P-value
heter / No heter	0 / 26	80 / 1100	0	1

(b) LPS2

	Opposite Direction	Same Direction	Fold Change	P-value
heter / No heter	5 / 21	77 / 1337	4.1	0.01

(c) LPS24
