# Supplementary material for: Joint genetic analysis using variant sets reveals polygenic gene-context interactions
Source: PLoS Genet. 2017 Apr 20;13(4):e1006693. doi: 10.1371/journal.pgen.1006693 (PMC5398484; doi:10.1371/journal.pgen.1006693)
Supplement: S7 Table — Shown is the computational complexity of iSet for alternative designs (either complete or stratified data designs), strategies to adjust for confounding (either principal components or a random effect) and analysis settings (number of variants R lower than the number of individuals N or not). Here, N denotes the number of individuals, R is the number of variants in the region, NPC is the number of principal components and t is the number of function evaluations of the optimizer. Operations that do not include t as factor refer to the cost of upfront computations that do not need to be evaluated in every step of the optimization. Operations that entail substantial computational burden (cubic operation in N) are highlighted in red. (PDF) [file pgen.1006693.s008.pdf]

Design	Strategy to correct for conf.	Complexity ($R < N$)	Complexity ($R > N$)
Complete	Principal components	$O(NR^2 + tNN_{\mathrm{PC}}^2)$	$O(N^3 + tNN_{\mathrm{PC}}^2)$
Stratified	Principal components	$O(tNR^2 + tNN_{\mathrm{PC}}^2)$	$O(tN^3)$
Complete	Random effect	$O(N^3 + N^2R + tNR^2)$	$O(tN^3)$
Stratified	Random effect	$O(tN^3)$	$O(tN^3)$
