# Supplementary figures and images for: Joint genetic analysis using variant sets reveals polygenic gene-context interactions
Source: PLoS Genet. 2017 Apr 20;13(4):e1006693. doi: 10.1371/journal.pgen.1006693 (PMC5398484; doi:10.1371/journal.pgen.1006693)

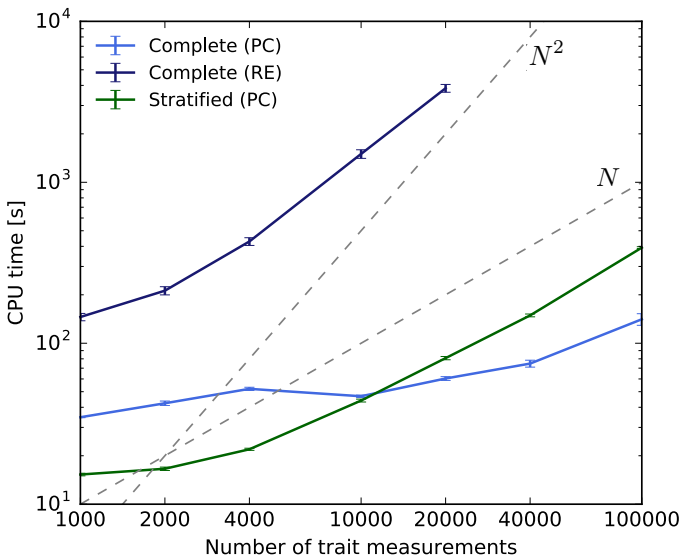

Supplement: S1 Fig — Shown is the average CPU time (in seconds) for one in interaction set test using a 30kb region for alternative designs and cohort sizes. By default iSet uses principal components to adjust for confounding (PC). Alternatively, iSet can be combined with an additional random-effect (RE) to adjust for structure such as relatedness (see Methods). Reported CPU times are empirical averages to perform the tests for association (mtSet), interaction (iSet) and heterogeneity-GxC (iSet-het) and include the cost of 30 parametric boostraps for each test to estimate P values (Methods). CPU times are averaged across 100 genomic regions (with a size of 30 kb, S1 Text). Note that the reported costs do not include the up-front cost of the principal components and the up-front eigenvalue decomposition of the global relatedness matrix for the random-effect method. Runtime estimates were obtained using a single core of an Intel Xeon CPU E5-2670 2.60 GHz processor. (PDF) [file pgen.1006693.s009.pdf]

**a** Analysis of complete designs

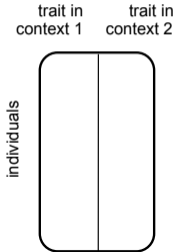

**b** Analysis of stratified samples from GWAS cohorts

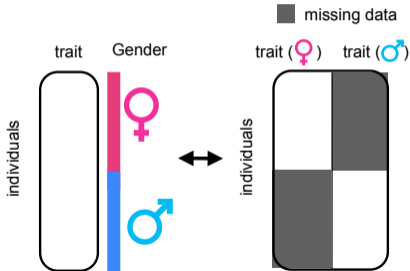

Supplement: S2 Fig — iSet supports efficient interaction set tests both for complete designs (where each individual is phenotyped in all analyzed contexts, (a), and stratified cohorts (where each individual is phenotyped in only one of the analyzed contexts, (b). (PDF) [file pgen.1006693.s010.pdf]

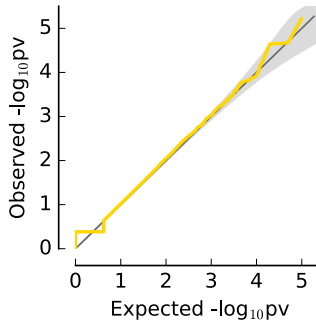

**(a)**  $\text{resc}=0.3$  -  $\text{nSNPs}=1$

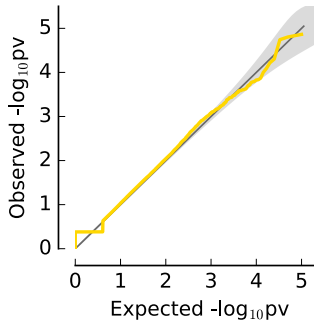

**(b)**  $\text{resc}=0.3$  -  $\text{nSNPs}=4$

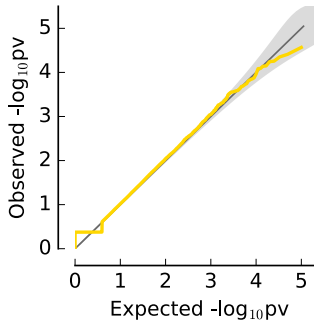

**(c)**  $\text{resc}=-0.5$  -  $\text{nSNPs}=4$

Supplement: S3 Fig — Shown is the QQ plot for the P values obtained from the heterogeneity-GxC interaction test (iSet-het) when simulating rescaling-GxC (without heterogeneity-GxC, Methods) for three different scenarios: (a) positive proportionality factor of effect sizes (0.3) and 1 causal variant, (b) positive proportionality factor of effect sizes (0.3) and 4 causal variants, (c) negative proportionality factor of effect sizes (-0.5) and 4 causal variants. (PDF) [file pgen.1006693.s011.pdf]

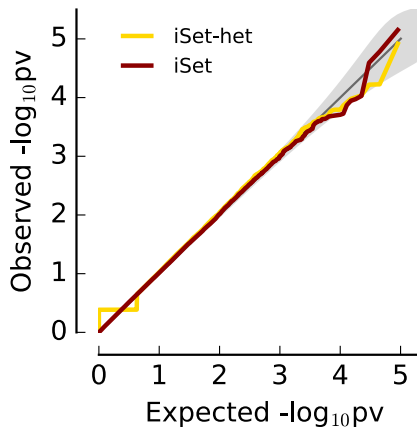

(a) 1 causal

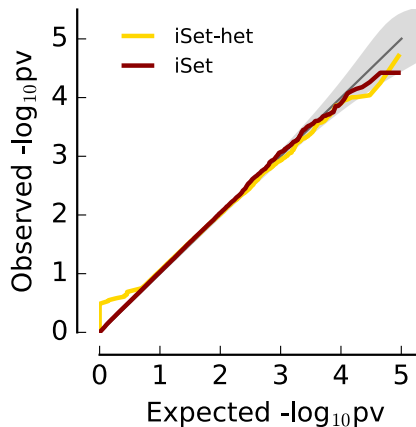

(b) GxG

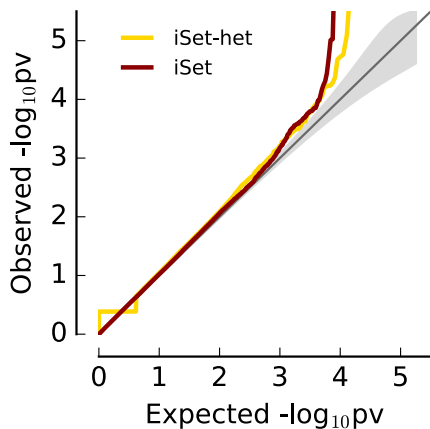

(c) Outlying samples - unnormalized

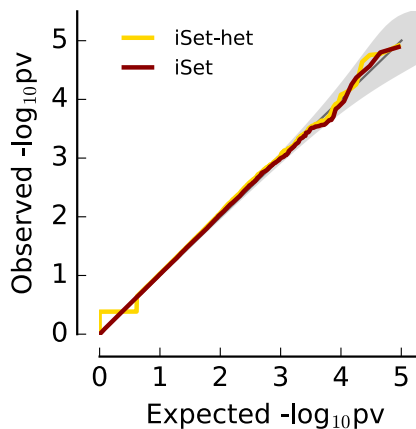

(d) Outlying samples - normalized

Supplement: S4 Fig — Shown are the QQ plots for the P values obtained from the interaction set test (iSet) and the heterogeneity-GxC set test (iSet-het) when simulating different types of model mismatch. (a, b) Simulated phenotypes that violate the assumption of the infinitesimal model iSet is based on (which assumes an additive linear model of all variants in the set): i) a single causal variant in the region (a) and ii) epistatic interactions (GxG, b, see Methods). (c, d) Violations of Gaussian distributed residuals, by simulating outlying samples (Methods). Notably, while iSet and iSet-het using unnormalised phenotypes yield inflated P values (c), both methods are well calibrated after quantile normalization to a unit variance Gaussian distribution (d, Methods). All the results from the real data applications are based on quantile normalized phenotypes (Methods). (PDF) [file pgen.1006693.s012.pdf]

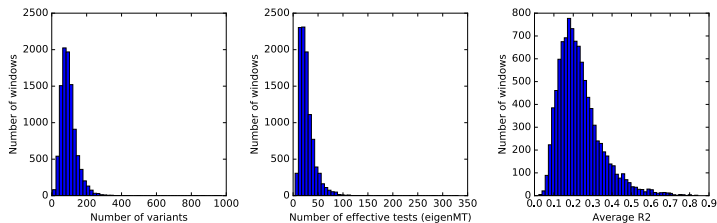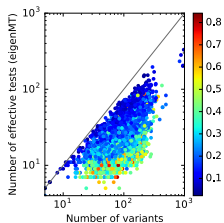

(a) 1000G

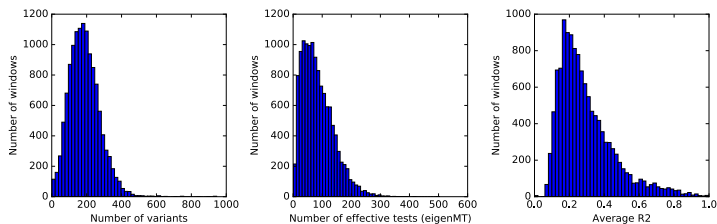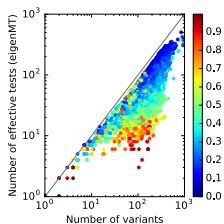

(b) Stimulus eQTL dataset

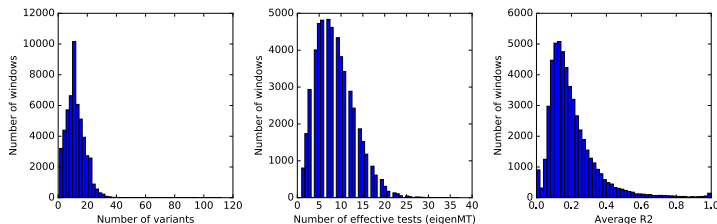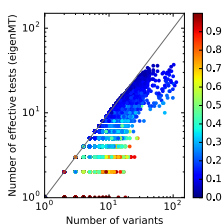

(c) NFBC1966

Supplement: S5 Fig — From left to right: distribution of the number of variants across the analyzed regions; distribution of the number of effective tests as estimated by eigenMT; distribution of the average pairwise squared Pearson correlation (r2) across all variants in each region; scatter plot of the number of effective tests versus the number of variants. Shown in color is the within-region average correlation (r2) across all pairs of variants. From top to bottom: (a) 10,000 30kb regions from the simulated data based on 1000 Genomes individuals, (b) 100kb cis regions (centered on the TSS) considered in the cis stimulus eQTL analysis (288 individuals) and (c) 100kb regions considered in genotype-sex interaction analysis in the NFBC1966 cohort (5,402 individuals). (PDF) [file pgen.1006693.s013.pdf]

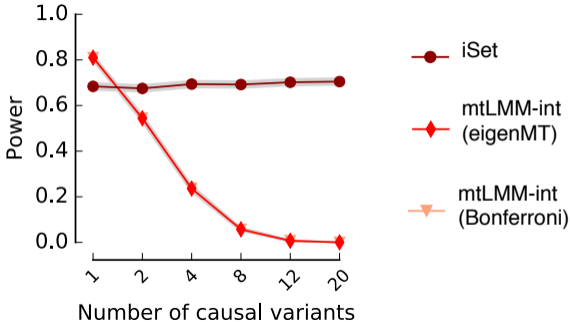

Supplement: S6 Fig — Shown are results analogous to those presented in Fig 2B, however, considering synthetic genotypes without LD. (PDF) [file pgen.1006693.s014.pdf]

**a**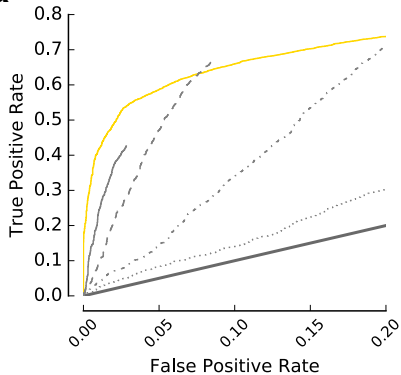**b**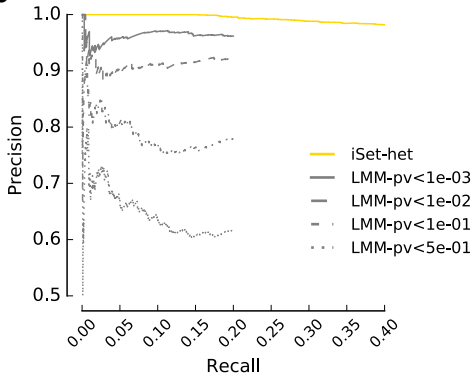

Supplement: S7 Fig — Receiver operating curve (a) and precision-recall curve (b) for alternative approaches to classify heterogeneity-GxC. Considered was the iSet test to score the extent of heterogeneity (iSet-het) and a baseline approach based on single-trait single-variant LMMs. Briefly, for the baseline model the considered score is defined as 1 − r2 (where r is the Pearson correlation coefficient between lead variants identified in each context) for regions with significant associations in both contexts (P-value thresholds 0.5, 0.01, 1e-3, 1e-4). Regions that were not marginally significant in either of the two contexts were assigned a score of zero (Methods). (PDF) [file pgen.1006693.s015.pdf]

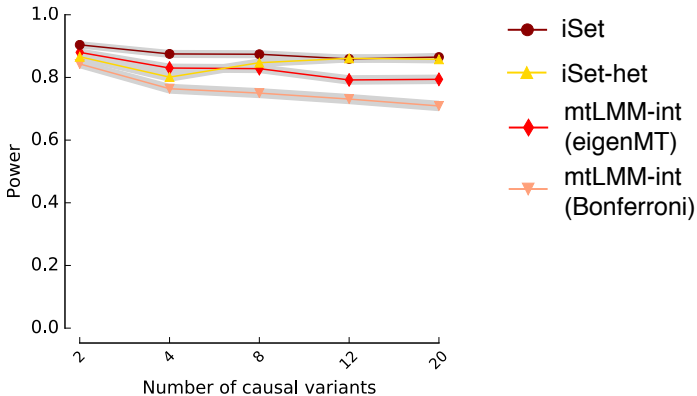

Supplement: S8 Fig — Shown is the power of iSet, iSet-het and a single-variant interaction test (mtLMM-int) for detecting GxC effects when simulating heterogeneity-GxC for increasing numbers of causal variants (0 < r < 0.8, where r is the correlation of the simulated genetic effects in the two contexts). (PDF) [file pgen.1006693.s016.pdf]

# GxE test

# het-GxE test

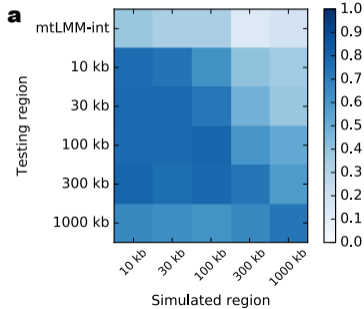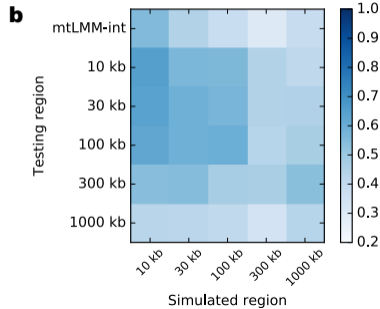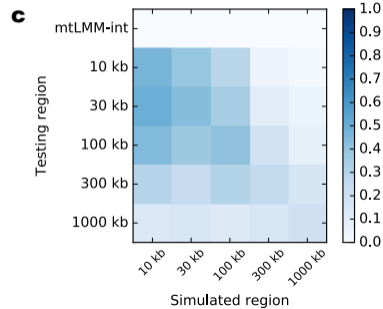

Supplement: S10 Fig — (a,b) Power of iSet when simulating causal regions of different sizes (x-axis) and for sliding window analyses with increasing window sizes (y-axis) (see Methods). Considered were both settings with pure rescaling (a) and settings with heterogeneity-GxC effects (b). For comparison, we also considered the power of mtLMM-int (adjusted for multiple testing within the same windows using eigenMT, Methods). (c) Power of iSet in the same settings as considered in (b) when simulating heterogeneity-GxC effects and for the iSet-het test. Note that mtLMM-int is not specific to detecting heterogeneity-GxC; hence the reported power was set to zero. (PDF) [file pgen.1006693.s018.pdf]

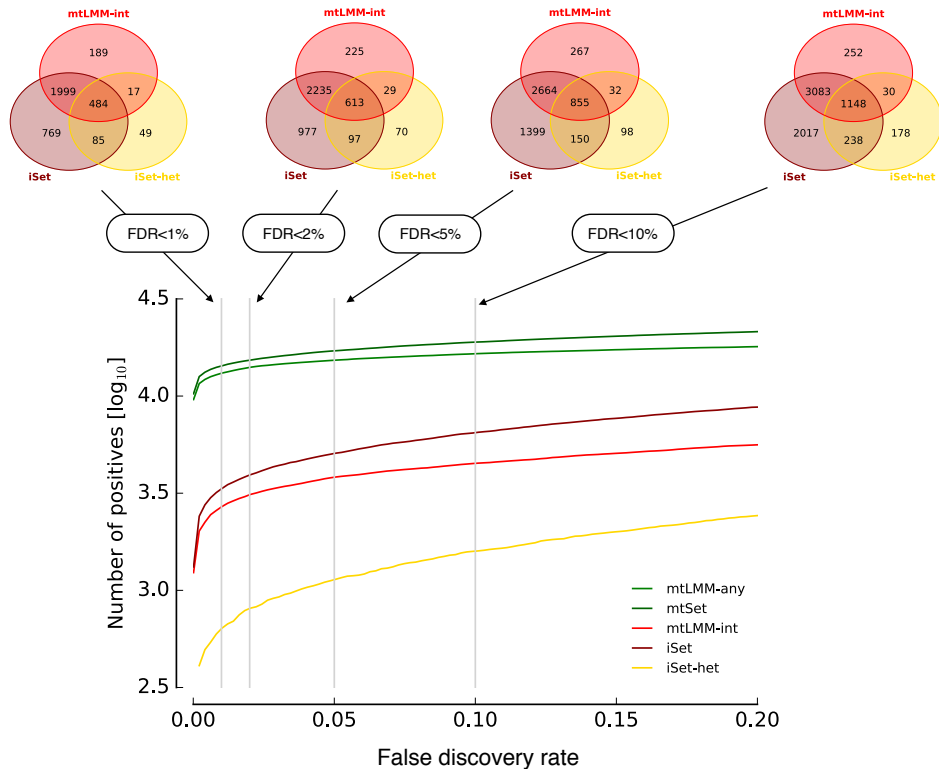

Supplement: S11 Fig — Shown is the number of probe/stimulus pairs with significant effects, detected by alternative single-variant and set-based tests, varying the genome-wide false discovery rate (FDR) threshold. Considered were the single-variant tests for associations (mtLMM-any) and interactions (mtLMM-int), as well as set tests for associations (mtSet), interactions (iSet) and heterogeneity-GxC effects (iSet-het). Venn diagrams on top show the overlap of significant probe/stimulus pairs for the three interaction tests at selected FDR thresholds (FDR<1%,2%,5%,10%). (PDF) [file pgen.1006693.s019.pdf]

IFN / naive

LPS2 / naive

LPS24 / naive

Association

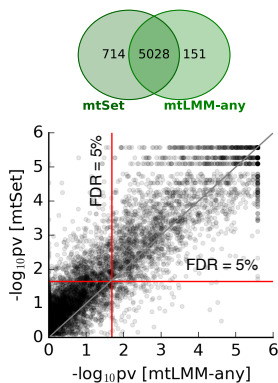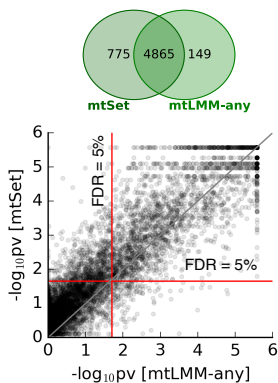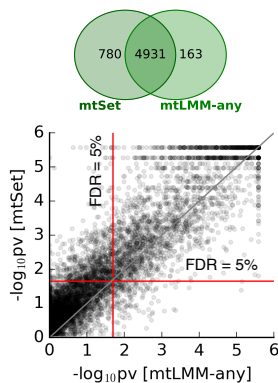

Interaction

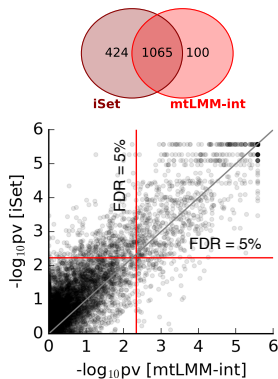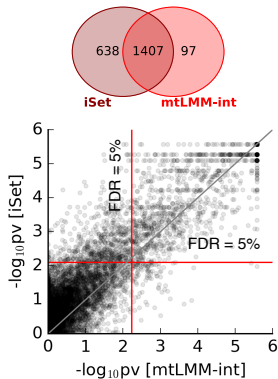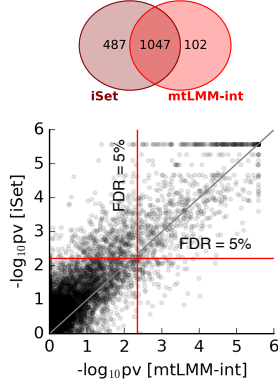

Supplement: S12 Fig — Shown are the scatter plots of the −log10P values from single-variant LMMs and set tests for association tests (mtLMM-any vs mtSet) and interaction tests (mtLMM-int vs iSet) for different stimulus contexts (IFN/naive, LPS-2h/naive, LPS-24h/naive). P values for single-variant models correspond to the minimum P-value across variants in the considered testing region, adjusted for the effective number of tests (estimated using eigenMT, Methods). Venn diagrams on the top of individual panels show the overlap of probes with significant associations or interactions identified using alternative methods (5% FDR). (PDF) [file pgen.1006693.s020.pdf]

**a**

OAS1 (chrom 12)

 $P_{\text{het}} = 5.27\text{e-}08$ 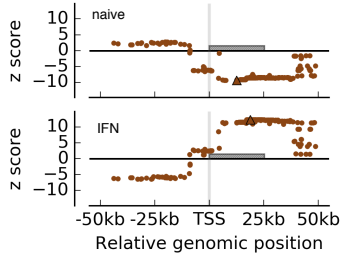**b**

LMNA (chrom 1)

 $P_{\text{het}} = 1.35\text{e-}04$ 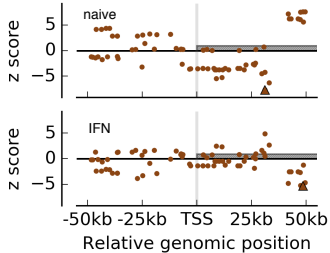**c**

PTK2B (chrom 8)

 $P_{\text{het}} = 7.31\text{e-}04$ 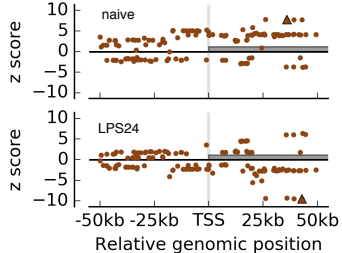

Supplement: S14 Fig — Shown is the z-score statistics for cis variants for OAS (a), LMNA (b) and PTK2B (c) across the contexts showing opposite effects. While the three examples are identified as opposite effects when using single-variant methods (Fairfax et al., Science, 2014), iSet identified significant heterogeneity-GxC, suggesting changes in the configuration of causal variants. Lead variants in individual contexts are annotated using triangles and are in high LD (r2 > 0.8). (PDF) [file pgen.1006693.s022.pdf]

**a**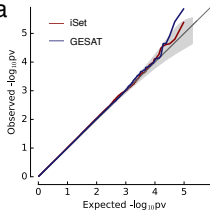**b**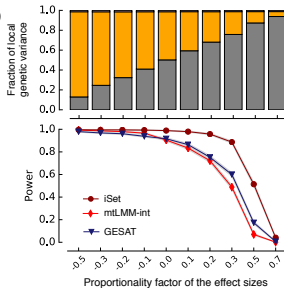**c**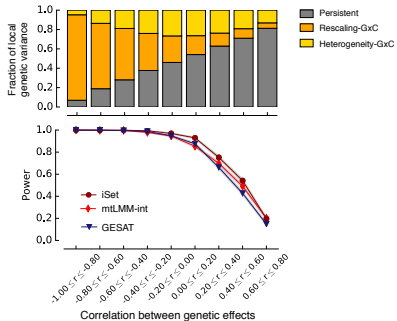

Supplement: S15 Fig — (a) QQ plot for the P values obtained when applying iSet and GESAT to synthetic datasets where only persistent genetic effects (No GxC) were simulated. (b) Power comparison of alternative interaction tests and variance decomposition results from iSet when simulating rescaling-GxC effects, for different factors of proportionality of the variant effect sizes across contexts. Considered were iSet, GESAT and a single-variant interaction test (mtLMM-int). (c) Analogous results as in b when simulating general GxC effects (both rescaling-GxC and heterogeneity-GxC). Results are stratified by the correlation between the simulated genetic effects between the two contexts. (PDF) [file pgen.1006693.s023.pdf]

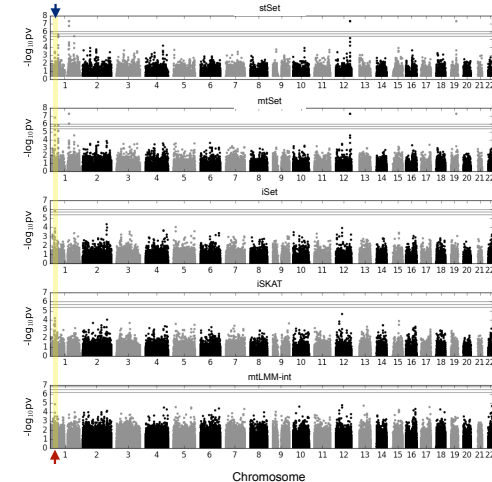

(a) crp3dec

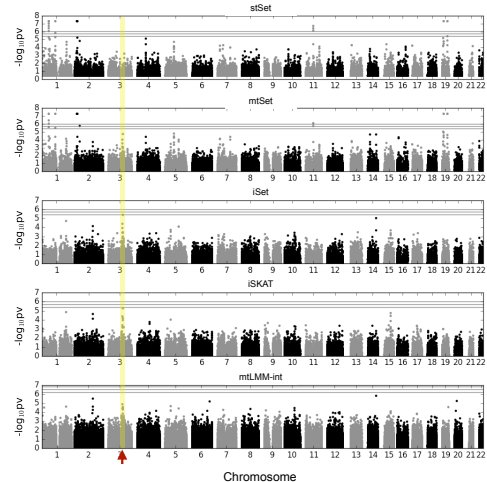

(b) FS\_KOL\_L

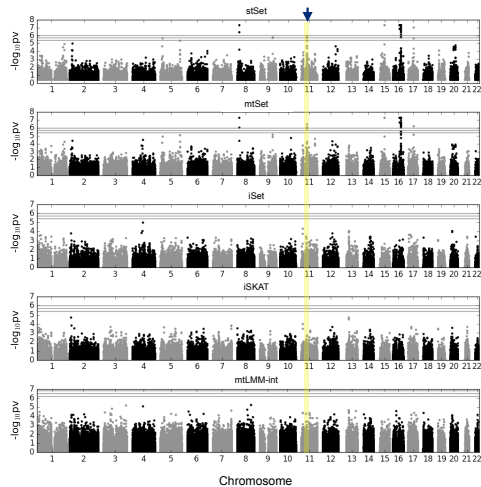

(c) FS\_KOL\_H

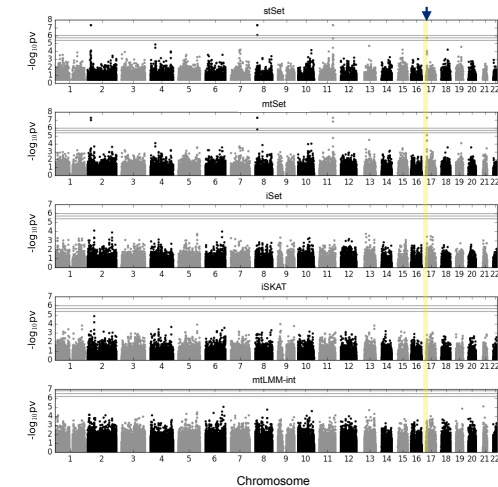

(d) FS\_TRIG

Supplement: S16 Fig — Shown are Manhattan plots for C-reactive protein (crp3dec, a), LDL cholesterol (FS_KOL_L, b), HDL cholesterol (FS_KOL_H, c), and triglycerides (FS_TRIG, d) obtained from univariate set tests ignoring sex-specific differences (stSet), an association test that accounts for differences in genetic effect across strata (mtSet), iSet, GESAT and single-variant interaction test (mtLMM-int). Red arrows indicate the interaction effects that are discussed in the main text. Blue arrows indicated associations that can only be detected when modeling differences in effect sizes across strata (mtSet vs stSet). (PDF) [file pgen.1006693.s024.pdf]

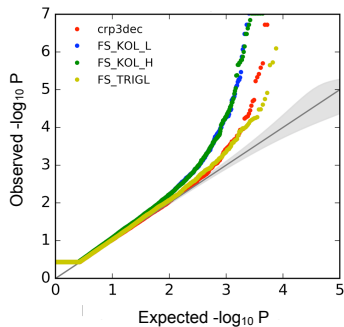

(a) stSet

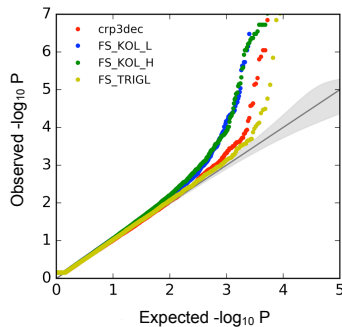

(b) mtSet

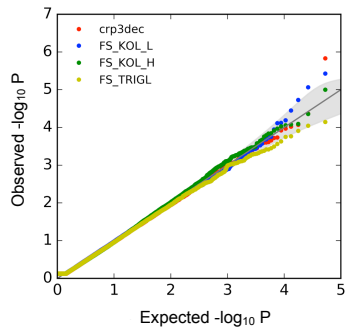

(c) iSet

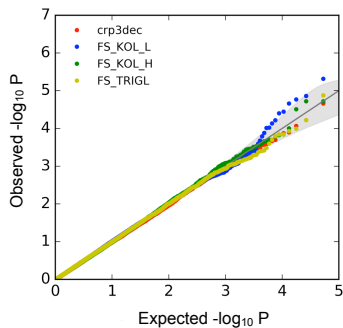

(d) GESAT

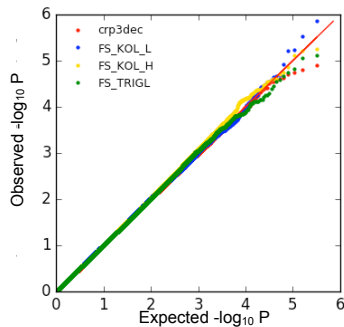

(e) mtLMM-int

Supplement: S17 Fig — Shown are the QQ plots for C-reactive protein (crp3dec), LDL cholesterol (FS_KOL_L), HDL cholesterol (FS_KOL_H), and triglycerides (FS_TRIG) obtained using a univariate association set tests ignoring sex (stSet, a), an association test modeling sex-specific genetic effects (mtSet, b), iSet (c), GESAT (d) and single-variant interaction test (mtLMM-int). (PDF) [file pgen.1006693.s025.pdf]

chrom 1

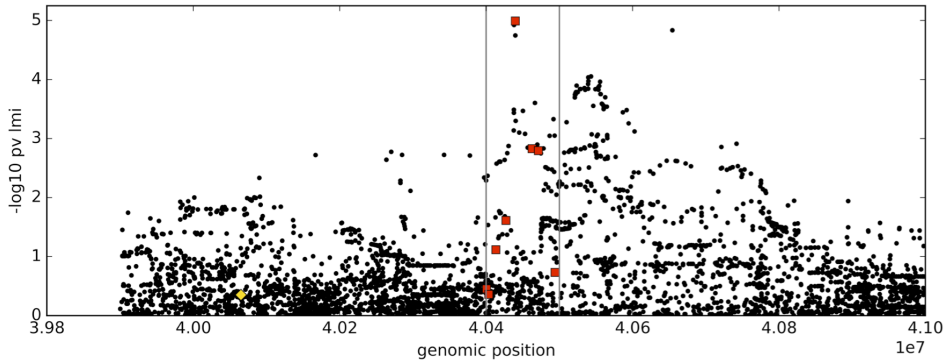

Supplement: S18 Fig — Shown is the Manhattan plot for C-reactive protein using single-variant interaction tests applied to common variants (MAF>0.5%) on imputed data. Vertical grey lines indicate the 100kb region with significant genotype-sex interaction (FWER = 10%) detected using iSet. Non-imputed typed variants are highlighted in red, showing that for this locus imputation strategies did not increase the power of single-variant methods. (PDF) [file pgen.1006693.s026.pdf]

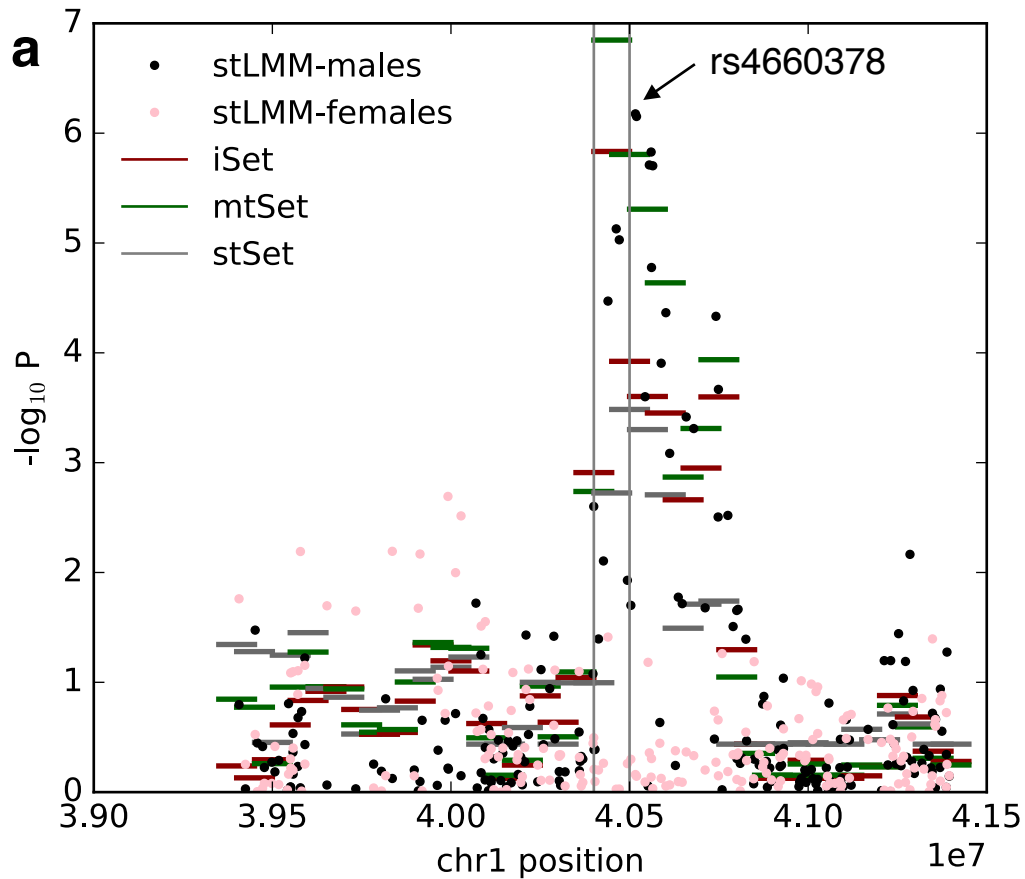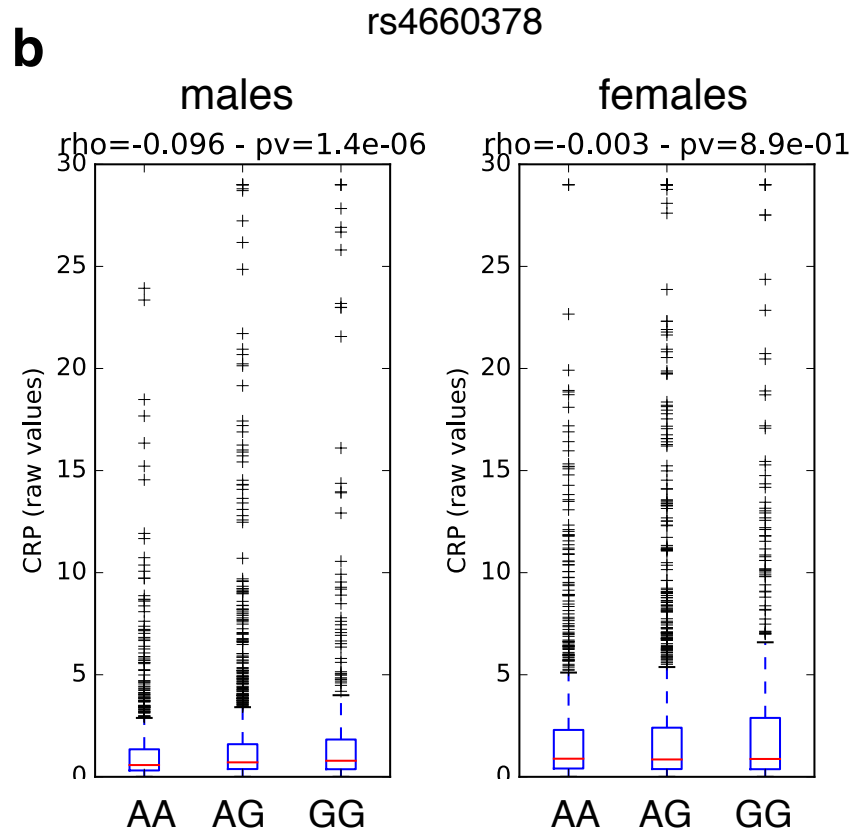

Supplement: S19 Fig — (a) Local Manhattan plot (1Mb around significant region) for single-variant association tests, either considering males (black) or females (pink). For comparison, shown are also the P values from the iSet (red), mtSet (green) and stSet (grey). (b) C-Reactive protein level stratified by different alleles of rs4660378 (lead SNP identified in the analysis using male individuals only). Rho in the caption correspond to the Spearman rank correlation coefficient and the corresponding P value, both for male and female individuals. (PDF) [file pgen.1006693.s027.pdf]
