# Supplementary material for: Joint genetic analysis using variant sets reveals polygenic gene-context interactions
Source: PLoS Genet. 2017 Apr 20;13(4):e1006693. doi: 10.1371/journal.pgen.1006693 (PMC5398484; doi:10.1371/journal.pgen.1006693)
Supplement: S9 Fig — Shown are the estimates of the genetic variance explained by the set component across all simulated settings when considering different covariance models. The variance component estimates are from a model that either considers a full-rank covariance (full, general case), a rank-one covariance (rank1, only rescaling-GxC) and a block covariance matrix (block, which models only persistent genetic effects). Both designs with fully observed cohorts (complete—1,000 individuals and 2 contexts for a total of 2,000 trait measurements) and stratified samples (stratified—2,000 individuals and 2 contexts for a total of 2,000 trait measurements) are considered. While the full-rank iSet model yields calibrated variance components, other methods yield biased estimates in some settings. In particular, we considered scenarios with either rescaling-GxC effects (where we varied the number of causal SNPs and the proportionality factor of the effect sizes across the two contexts) or heterogeneity-GxC (where we vary the number of SNPs). For each simulated scenario, we considered 1,000 simulated regions and altered the variance explained by the region (we consider the values 2%, 5% and 10%). Grey horizontal lines denote the true simulated local genetic variance. (PDF) [file pgen.1006693.s017.pdf]

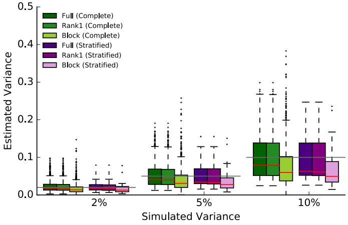

(a) Resc-GxC (0.3) - 1 causal

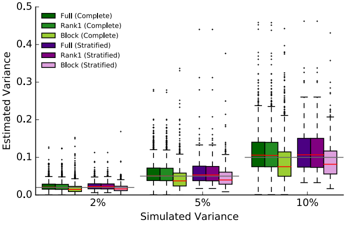

(b) Resc-GxC (0.3) - 4 causal

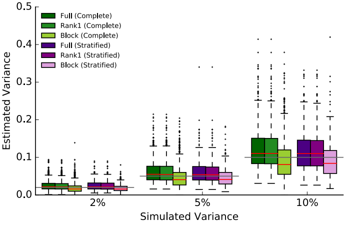

(c) Resc-GxC (0.3) - 8 causal

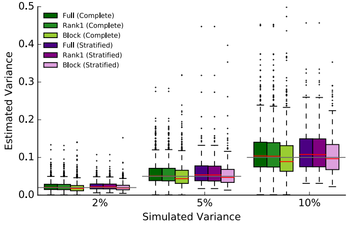

(d) Resc-GxC (0.5) - 4 causal

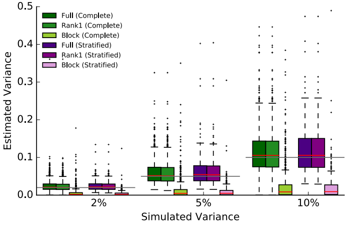

(e) Resc-GxC (-0.5) - 4 causal

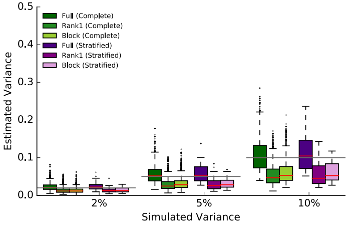

(f) Het-GxC - 2 causal

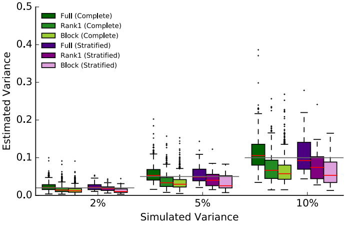

(g) Het-GxC - 4 causal

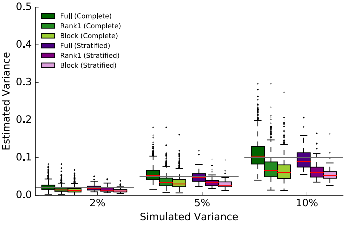

(h) Het-GxC - 8 causal
