# Supplementary material for: Joint genetic analysis using variant sets reveals polygenic gene-context interactions
Source: PLoS Genet. 2017 Apr 20;13(4):e1006693. doi: 10.1371/journal.pgen.1006693 (PMC5398484; doi:10.1371/journal.pgen.1006693)
Supplement: S13 Fig — (a) Scatter plot of the maximum Q values across pairs of jointly analyzed contexts (independent analysis), comparing set tests and single-variant tests for different stimulus pairs. Heterogeneity-GxC cases without clear single-variant interpretation (highlighted in red) tend to be associated with increased significance when using set tests, suggesting that differences in power may impair the single-variant annotation of these eQTLs. (b) Histogram of the squared Pearson correlation (r2) of cis genetic effects predicted using the best linear unbiased predictor, BLUP (Methods) across pairs of contexts. Shown is the distributions either for all probes with significant effects in the naive context and probes with significant heterogeneity-GxC. As expected, genes with heterogeneity-GxC tend to be associated with lower correlation of genetic effects across contexts. (c) Scatter plot of the correlation coefficient of genetic effects (as in b) versus the maximum q-value across the naive and the stimulated state. Heterogeneity eQTLs (highlighted in red) tend to have stronger marginal associations and lowly-correlated cis genetic signals. (PDF) [file pgen.1006693.s021.pdf]

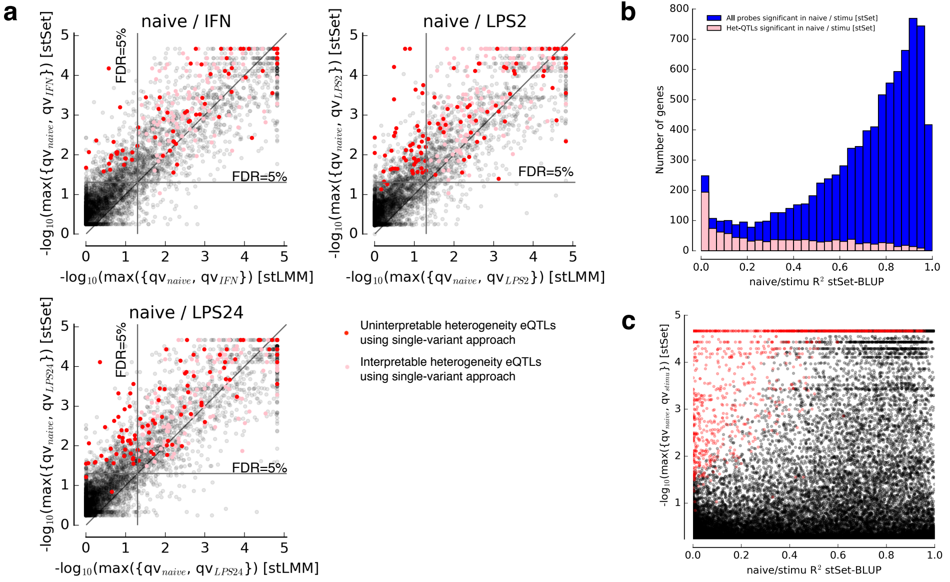
a
naive / IFN
naive / LPS2
naive / LPS24
FDR=5%
$-\log_{10}(\max(\{qv_{naive}, qv_{IFN}\})$ [stLMM]
$-\log_{10}(\max(\{qv_{naive}, qv_{IFN}\})$ [stSet]
$-\log_{10}(\max(\{qv_{naive}, qv_{LPS2}\})$ [stLMM]
$-\log_{10}(\max(\{qv_{naive}, qv_{LPS2}\})$ [stSet]
$-\log_{10}(\max(\{qv_{naive}, qv_{LPS24}\})$ [stLMM]
$-\log_{10}(\max(\{qv_{naive}, qv_{LPS24}\})$ [stSet]
Uninterpretable heterogeneity eQTLs using single-variant approach
Interpretable heterogeneity eQTLs using single-variant approach
b
All probes significant in naive / stimu [stSet]
Het-QTLs significant in naive / stimu [stSet]
Number of genes
naive/stimu R^2 stSet-BLUP
c
$-\log_{10}(\max(\{qv_{naive}, qv_{stimu}\})$ [stSet]
naive/stimu R^2 stSet-BLUP
